# The interplay between climatic niche evolution, polyploidy and reproductive traits explains plant speciation in the Mediterranean Basin: a case study in *Centaurium* (Gentianaceae)

**DOI:** 10.3389/fpls.2024.1439985

**Published:** 2024-08-09

**Authors:** Ana Valdés-Florido, Virginia Valcárcel, Enrique Maguilla, Zoila Díaz-Lifante, Cristina Andrés-Camacho, Louis Zeltner, Marina Coca-de-la-Iglesia, Nagore G. Medina, Juan Arroyo, Marcial Escudero

**Affiliations:** ^1^ Department of Plant Biology and Ecology, Faculty of Biology, University of Seville, Seville, Spain; ^2^ Departamento de Biología, Universidad Autónoma de Madrid, Madrid, Spain; ^3^ Centro de Investigación en Biodiversidad y Cambio Global (CIBC-UAM), Universidad Autónoma de Madrid, Madrid, Spain; ^4^ Department of Molecular Biology and Biochemical Engineering, Pablo de Olavide University, Seville, Spain; ^5^ Laboratoire de Botanique Evolutive, Université de Neuchâtel, Neuchâtel, Switzerland

**Keywords:** centauries, climatic niche, chromosome evolution, Mediterranean climate, phylogeny, polyploidy, speciation

## Abstract

Speciation and diversification patterns in angiosperms are frequently shaped by niche evolution. *Centaurium* Hill is a Mediterranean genus with ca. 25 species, of which 60% are polyploids (tetra- and hexaploids), distributed mainly in the Mediterranean Basin and in areas with temperate and arid climates of Asia, Europe, North-Central Africa and North America. The evolutionary history of this genus has been studied using morphological, biogeographical and molecular approaches, but its climatic niche characterization and its relation with genome evolution (chromosome number and ploidy level) has not been addressed yet. Thus, this study aims to identify the role of the evolution of climatic niche, ploidy level, life cycle and floral traits in the diversification of *Centaurium*. Climatic niche characterization involved estimating present climate preferences using quantitative data and reconstructing ancestral niches to evaluate climatic niche shifts. The evolution of climatic niche towards selective optima determined by ploidy level (three ploidy levels) and different binary traits (polyploidy, floral size, floral display, herkogamy and life cycle) was addressed under the Ornstein-Uhlenbeck model. Chromosome number evolution was inferred using the ChromoSSE model, testing if changes are clado- or anagenetic. Chromosome number evolution and its link with cladogenesis, life cycle and floral traits was modeled on the phylogeny. The reconstruction of the ancestral niches shows that *Centaurium* originated in a mild climate and diversified to both humid and cold as well as to dry and warmer climates. Niche conservatism was estimated in the climatic niche of the ancestors, while the climatic niche of the current taxa experienced transitions from their ancestors’ niche. Besides, the evolution of climatic niche towards multiple selective optima determined by the studied traits was supported, life cycle optima receiving the highest support. The reconstruction of chromosome number transitions shows that the rate of speciation process resulting from chromosomal changes (chromosomal cladogenesis) is similar to that of non-chromosomal cladogenesis. Additionally, dependent evolution of floral size, floral display and herkogamy with chromosome number variation was supported. In conclusion, polyploidization is a crucial process in the Mediterranean region that assisted speciation and diversification into new areas with different climates, entailing niche shifts and evolution of reproductive strategies.

## Introduction

The crucial influence of climate in plant speciation has long been recognized (Hua and Wiens, 2013). Two of the mechanisms driving speciation are niche divergence and niche conservatism. On the one hand, niche divergence refers to the evolutionary process for which a species undergoes changes in its requirements (e.g. climatic requirements), adapting to new environmental conditions that differ from the original niche (Schluter, 2000) and leading ultimately to reproductive isolation (Schluter, 2001, 2009; Rundle and Nosil, 2005). On the other hand, under the niche conservatism hypothesis (Wiens, 2004) the species maintain similar tolerances over time. Niche conservatism has been suggested to foster speciation by supporting isolation of populations that may then undergo allopatric speciation (Wiens, 2004; Qiao et al., 2024).

Another mechanism that fosters speciation in angiosperms are chromosome rearrangements, and especially those mechanisms implying chromosome number variations (Stebbins, 1950; Grant, 1981; Soltis et al., 2009, 2015). A significant correlation between species richness and diversification rates with chromosome number variation has been inferred for the whole clade of the angiosperms (Carta and Escudero, 2023). These variations comprise the multiplication of the entire set of chromosomes (i.e., polyploidy) or changes in chromosome number resulting from fusion and fission (i.e., dysploidy) (Mayrose et al., 2010; Schubert and Lysak, 2011). The evolutionary significance of the different types of chromosome changes in terms of temporal persistence across evolution has long been debated for angiosperms. For example, there is evidence of several rounds of polyploidization across the angiosperm tree of life (Leitch and Bennett, 2004; Van de Peer et al., 2009; Wood et al., 2009; Jiao et al., 2011; Mayrose et al., 2011; Van de Peer, 2011; Escudero and Wendel, 2020), which suggests long-term persistence of polyploid lineages. Similarly, gains and losses of single chromosomes (dysploidy) are also widespread among angiosperms (Brochmann et al., 2004) and seem to persist even longer over time than polyploidy (Escudero et al., 2014). Interestingly, the majority of hyper-diverse genera in angiosperms originated after recurrent events of polyploidy (Landis et al., 2018). Whole genome duplications (WGD) provide genetic support for ecological differentiation and adaptation (Levin, 2002) and, as such, they may boost speciation and trigger diversification increases. Different cytotypes may have different tolerances to temperature, drought, salinity, and herbivory, as well as different pollinators and, subsequently, differences in floral structure (Levin, 2004). In line with this, some studies have evaluated whether whole genome duplication events are linked to climate niche shifts with some of them supporting niche divergence between polyploids and diploids (Theodoridis et al., 2013; Thompson et al., 2014) while others do not (Godsoe et al., 2013; Castro et al., 2019). Therefore, investigating in which instances WGD is associated with niche shifts can help clarify the role of polyploidy in evolutionary processes.

The evolution of flowering plants has also been modulated by the association with pollinators. The change from outbreeding to selfing is one of the most frequent evolutionary transitions in angiosperms (Stebbins, 1950, 1957, 1974; Barrett, 2002), and is usually accompanied by a set of morphological and functional changes of flowers, jointly termed selfing syndrome (Darwin, 1876; Ornduff, 1969; Richards, 1986). These morphological changes include the reduction in the distance between anthers and stigmas within the same flower (reduction of the degree of herkogamy) (Husband and Barrett, 1992; Barrett, 1993), the reduction of floral size (Goodwillie et al., 2010), or the reduction in floral display (Sicard and Lenhard, 2011), among others, with the latter two being related to the reduction of attraction of pollinators (Goodwillie et al., 2010). Besides, the evolution of selfing is promoted by an annual or short-lived life cycle, particularly in colonizer species due to their frequent need to endure a scarcity or dearth of pollinators (Barrett, 2010; Pannell, 2015).

**Figure 1:**
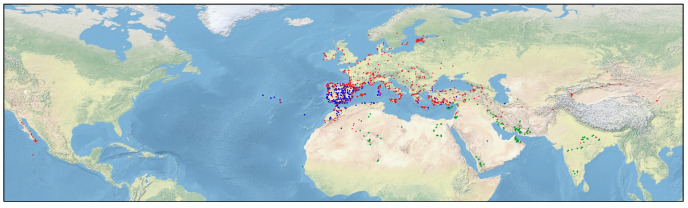
Distribution map of *Centaurium*. Blue circles represent occurrences of diploid taxa, red circles represent tetraploid taxa and green ones, hexaploid taxa.

Here, we focus on the genus *Centaurium* Hill (Gentianaceae), which comprises ca. 25 species (Mansion and Struwe, 2004; Díaz-Lifante, 2012; POWO, 2023). This genus is distributed around temperate and arid climates of Asia, Europe, North-Central Africa, and North America (**Figure 1**), although its center of diversification is the Mediterranean Basin (Mansion and Struwe, 2004; Maguilla et al., 2021). The taxonomy and morphology (particularly floral traits) of this genus has been widely studied (Zeltner, 1970; Mansion and Struwe, 2004; Díaz-Lifante, 2012; Jiménez-Lobato et al., 2019), and also some phylogenetic studies have been performed to date (Mansion and Struwe, 2004; Mansion et al., 2005; Jiménez-Lobato et al., 2019; Maguilla et al., 2021; Valdés-Florido et al., 2024). These previous studies confirmed generalized patterns of hybridization within the genus (Mansion and Struwe, 2004; Mansion et al., 2005; Guggisberg et al., 2006; Valdés-Florido et al., 2024), leading to complex morphological variability and thus, different taxonomic treatments (Mansion and Struwe, 2004; Mansion et al., 2005; Díaz-Lifante, 2012). Additionally, cytogenetic studies show that *Centaurium* is a polyploid complex in which different ploidy levels are found: diploid (2*x*), tetraploid (4*x*) and hexaploid (6*x*) (Zeltner, 1970). Recent studies have been accomplished to study the evolution of chromosome numbers in *Centaurium* (Maguilla et al., 2021; Escudero et al., 2023) using ChromEvol (Glick and Mayrose, 2014). The haploid chromosome number *x* = 10 was estimated as the most probable base chromosome number for the genus, although a secondary base chromosome number of *x* = 9 was also inferred (Escudero et al., 2023). Maguilla et al. (2021) and Escudero et al. (2023) showed that the main chromosome number transitions in *Centaurium* have been both descending dysploidy and polyploidy, with polyploidization estimated to mainly occur towards the tips of the phylogeny. Allopolyploidy (polyploidy involving hybridization) involved lineages of the two different base numbers (9 and 10), as previously reported by Zeltner (1970). Interestingly, ploidy levels follow a geographical pattern in *Centaurium*, where diploid species are distributed mainly in the Mediterranean Basin, tetraploids are found generally at higher latitudes in northern Europe and eastern Asia, and the hexaploids can be found at southern latitudes in India, the southwest of the Mediterranean Basin and the Arabian Peninsula (Mansion et al., 2005). In line with this, Maguilla et al. (2021) showed a strong relation of polyploidy with geographical movements. These results confirm that polyploidy occurred in species and subspecies that are distributed outside the area of origin (i.e., the Mediterranean Basin), suggesting that polyploidy may have facilitated range expansions by increasing establishment success in the newly colonized areas. Thus, polyploidization events do not seem to facilitate dispersal events *per se*, but rather the establishment in the new areas or under different niches (Maguilla et al., 2021).

The evolution of reproductive traits and its association with polyploidy has also been studied in *Centaurium* (Jiménez-Lobato et al., 2019). Specifically, traits associated with selfing syndrome were studied: herkogamy, floral size, floral display, and polyploidy. No pattern of correlated evolution was found in the genus: *Centaurium* evolved its selfing ability early in its history, while polyploidy and flower traits evolved subsequently, probably independently (Jiménez-Lobato et al., 2019).

Despite the great climatic variations of the area of distribution of *Centaurium* (from temperate and humid areas to warmer and dry ones), the relation of the large variability in morphological traits, life cycle (annual/biennial vs. perennial) and ploidy levels with the climatic niche has not been studied yet. In this context, we aim to (i) estimate the species’ climatic niche as inferred from occurrences and reconstruct its evolution, (ii) model chromosome evolution and cladogenesis, (iii) test the possible link between niche evolution and selective optima related to polyploidy and reproductive traits, and (iv) test the dependent or independent evolution of chromosome number with floral traits and life cycle. To do so, we used a comprehensive geographical and climate database, which included chromosome counts, data of morphological traits and life cycle of the species, as well as a phylogenetic reconstruction based on RADseq markers (Valdés-Florido et al., 2024). We hypothesize that changes in ploidy level are related to the climatic niche in *Centaurium*. Thus, we expect to find polyploids to have niches with more extreme climates, and diploids with niches of milder conditions. The null hypothesis expected from the models of climatic niche evolution regarding morphological traits and life cycle is that the evolution occurs toward a single optimum. Besides, we hypothesize that chromosome evolution played a role in cladogenetic events in *Centaurium* and expect to find independent evolution of floral traits and chromosome number changes, as suggested by previous studies (Jiménez-Lobato et al., 2019).

## Materials and methods

### Occurrence records

This study included 28 *Centaurium* taxa (see **Supplementary Table S1**), following the taxonomic treatment proposed by Mansion et al. (2005) and then reviewed by Díaz-Lifante (2012). The occurrence records were obtained from different herbaria: University of Seville (SEV), University of Oviedo (FCO), Royal Botanic Gardens of Madrid (MA), University of Santiago de Compostela (SANT), University of Valencia (VAL), University of Málaga (MGC) and University of Neuchâtel (NEU), the flora of the Iberian Peninsula project (*Flora Iberica*, Díaz-Lifante, 2012), and from our field sampling. To fill potential gaps in taxa distribution, GBIF data was also downloaded (https://www.gbif.org/es/). The original database contained 3,413 records.

To ensure the taxonomic and geographic quality of the database, a filtering process similar to that described by Coca-de-la-Iglesia et al. (2023) was conducted. This procedure includes a first step of data cleaning: removing records that lack coordinates, the ones that have invalid coordinates (i.e., latitude or longitude = 0), those in which coordinates have low precision (i.e., the coordinates that had fewer than 2 decimals), as well as duplicates. Given that the original databases included records from GBIF, which may increase taxonomic uncertainty due to species being incorrectly identified, the Coca-de-la-Iglesia et al.’s procedure includes filtering steps that consist of cross-referencing records with political countries describing the natural distribution of taxa according to Díaz-Lifante (2012). Subsequently, maps were built and visually inspected to double-check the natural distribution of each taxon and evaluate spatial gaps per taxon (**Supplementary Figure S2**). Country codes were assigned using the Level-3 code of the World Geographical Scheme for Recording Plant Distributions (WGSRPD) from the Biodiversity Information Standards (TDWG) (http://www.tdwg.org/standards/109). Records falling outside the natural distribution of the species were removed. The third and final cleaning step included recalculating the off-land coordinates so they fell in the nearest terrestrial climatic cell of the template (Coca-de-la-Iglesia et al., 2023). The records located more than 5 km from the coastal limit were removed. As a result, we compiled a clean database with 1,690 records.

### Climatic niche characterization

We extracted 19 bioclimatic variables from WorldClim (Hijmans et al., 2005) with a resolution of 30 seconds. To avoid collinearity effects, pairwise Pearson correlation was calculated among the bioclimatic variables. The correlation values were transformed into a dendrogram to visualize the results (**Supplementary Figure S2**). We then removed correlated variables and selected those below 0.5 using R (R Core Team, 2023) that had sound biological interpretation for our species. The subsequent climatic analyses were performed using four variables representing temperature (bio1 -annual mean temperature-, bio2 -mean diurnal range-, bio7 -temperature annual range-, bio8 -mean temperature of wettest quarter-) and three variables representing precipitation (bio12 -annual precipitation-, bio15 -precipitation seasonality-, and bio17 -precipitation of driest quarter-). A Principal Component Analysis (PCA) was performed using the function *dudi.pca* from the R package *ade4* (Dray and Dufour, 2007), with the least correlated climatic variables for the studied *Centaurium* taxa.

### Climatic niche reconstruction analyses

Climatic niche reconstruction analyses were performed using the phylogeny of the genus of Valdés-Florido et al. (2024) as the input tree. The original phylogeny was reconstructed using a maximum likelihood approach based on a SNP matrix consisting of 8,497 SNPS out of 215,894 total recovered loci derived from RADseq markers, comprising the same 28 taxa (Valdés-Florido et al., 2024). We pruned the original phylogeny to keep only one tip per taxon for the climatic niche reconstruction using the R package *ape* (Paradis and Schliep, 2019). Most taxa (species and subspecies) were monophyletic, but two taxa were polyphyletic (*C. littorale* ssp. *uliginosum* and *C. somedanum*) and one was paraphyletic (*C. scilloides*). As there was no spatial or morphological pattern to deal with polyphyletic taxa, we randomized the removal of tips for these three non-monophyletic taxa.

As climate input data we used the first axis of the PCA (PC1), as it was the principal component that better explains the data variability. To include the current climatic heterogeneity of each taxon in the ancestral reconstruction, we used the probabilistic approach developed in Coca-de-la-Iglesia et al. (2024). This probabilistic approach consists of generating 1,000 climatic matrices from the original species PC1 values as proportional to the climatic density function of each species. Then, this procedure uses the *fastAnc* function from *phytools* 1.5-1 R package (Revell, 2012) with 1,000 iterations, performing a fast estimation of the ancestral states following a Maximum Likelihood approach, under a Brownian motion (BM) model. Finally, for each of the ancestral nodes this procedure summarizes the climatic density function.

### Evolution of climatic niche regarding polyploidy, life cycle and morphological traits

We used the Ornstein-Uhlenbeck (OU) model (Hansen, 1997; Blomberg et al., 2020) to test the evolution of climatic niche towards selective optima determined by ploidy levels (three optima: 2*x*, 4*x* and 6*x*) and different binary traits (two optima): polyploidy (diploids vs. polyploids), floral size (small to medium vs. large), floral display (low vs. high), herkogamy (low herkogamy vs. high herkogamy) and life cycle (annual/biennial vs. perennial). To conduct these analyses, we used the phylogenetic tree previously employed for climatic niche reconstruction. The binary trait characterization used (polyploidy, floral size, floral display, herkogamy and life cycle data) was that from Jiménez-Lobato et al. (2019) (**Supplementary Table S1**). The continuous variables were the climatic ones used in the climatic niche reconstruction. The null hypothesis is that in the evolutionary history of the genus there is only a single selective optimum for the studied traits. Conversely, the alternative hypothesis suggests the presence of multiple optima. To test the evolution of climatic optima determined by the three ploidy levels (2*x*, 4*x* and 6*x*), we followed two steps. First, we designed a transition matrix allowing only unidirectional shifts from 2*x* to 4*x* and from 4*x* to 6*x*, to estimate the model of ploidy evolution and map the ancestral states in the phylogeny. The model was fitted using the *fitDiscrete* function from the R-package *geiger* (Harmon et al., 2008; Pennell and Harmon, 2013) and the stochastic mapping was performed with the *make.simmap* function from the R-package *phytools* (Revell, 2012) under a “all rates different” (ARD) model for reproductive traits and life cycle. Second, we estimated the climatic optima based on the three ploidy levels using the Ornstein-Uhlenbeck model. A similar approach was used for the binary trait diploid vs. polyploid. The evolution of the climatic niche toward selective optima for the morphological traits, life cycle and three-state ploidy levels was tested using the *OUwie* function from the R-package *OUwie* (Beaulieu et al., 2012; Beaulieu and O’Meara, 2020), under the “OUM” model. Besides, the null hypothesis was tested under the “OU1” model of the same R function. We used the Akaike information criterion (AIC) with the R package *diversitree* (FitzJohn, 2012) to select the best-fitting model.

### Clado- and anagenetic chromosome evolution in *Centaurium*

The ChromoSSE model (Freyman and Höhna, 2018), implemented in the RevBayes platform (Höhna et al., 2016), was used to infer the chromosome number evolution along the phylogeny of the genus and to test if these changes were either clado- or anagenetic. The used input tree was the pruned tree used for the climatic reconstruction, and the chromosome counts were recovered from Maguilla et al. (2021). The ChromoSSE model consisted of cladogenetic parameters (no change, fission, fusion and polyploid events), a global rate of extinction and anagenetic parameters (fission, fusion and polyploidization events). Here, we set the demi-polyploidy parameter to 0, as no demi-polyploidization event was inferred in previous studies (Maguilla et al., 2021; Escudero et al., 2023). We then visualized the results using the RevGadgets R package (Tribble et al., 2022).

### Dependent vs. independent model of trait evolution and chromosome number

The dependent evolution between chromosome number evolution and life cycle (annual/biennial vs. perennial) and reproductive traits (floral size, floral display and herkogamy), respectively, was studied with the R package *ChromePlus* (Blackmon et al., 2019). This approach infers a different model of chromosome evolution for each state of the binary trait under study. The used phylogeny was the one used for the climatic niche reconstruction, the chromosome counts were recovered from Maguilla et al. (2021), and the floral traits and life cycle characterization from Jiménez-Lobato et al. (2019). We used the R package *diversitree* (FitzJohn, 2012) to compare the AIC of a model of dependent evolution of each of the binary traits and chromosome evolution against a model of independent evolution. The estimated parameters included transitions (tran01 and tran10) and chromosome parameters: chromosome gain (gain0 and gain1), chromosome loss (loss0 and loss1) and polyploidy (polyploidy0 and polyploidy1).

## Results

**Figure 2:**
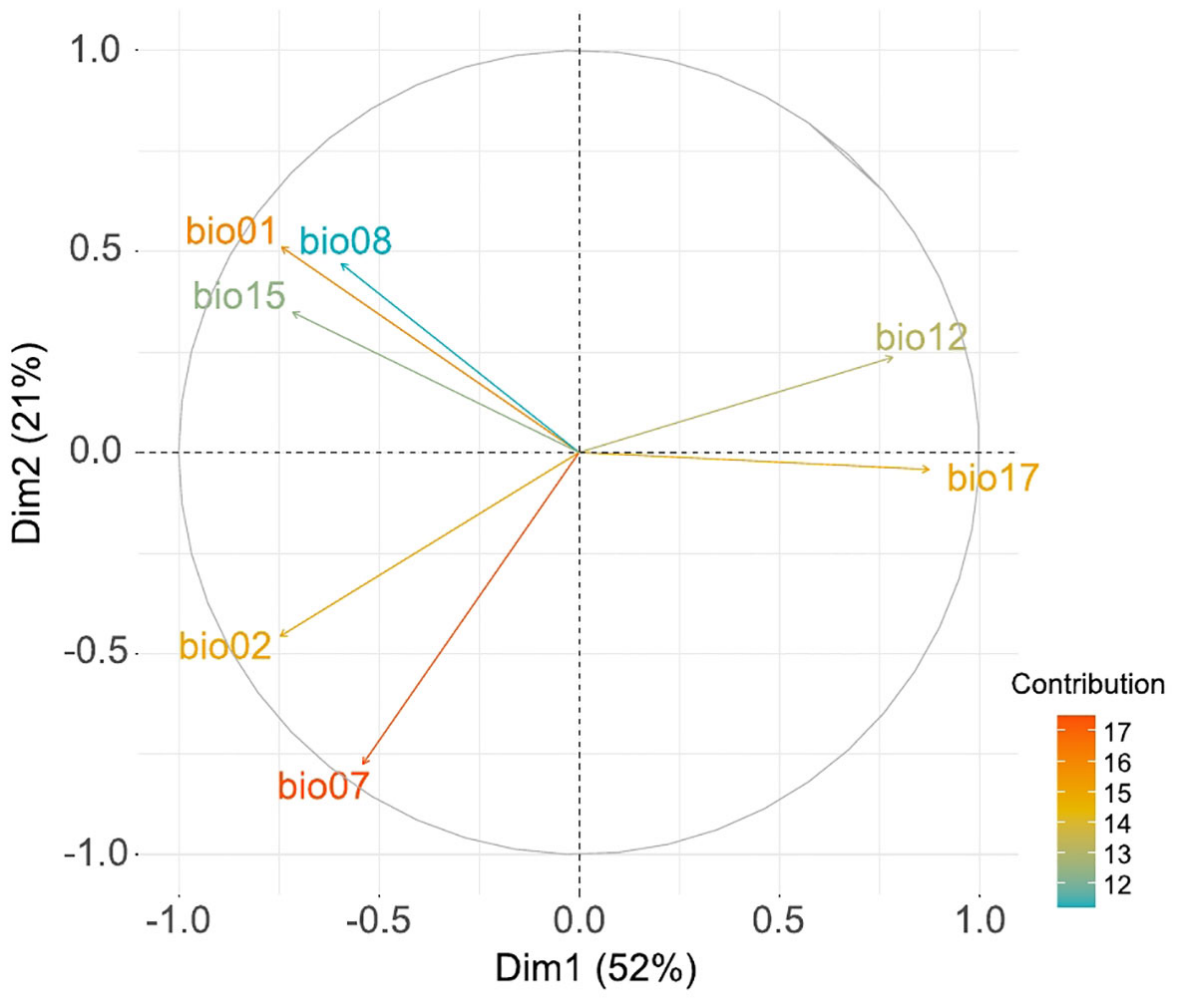
Principal Component Analysis performed with the selected bioclimatic variables: bio01 (annual mean temperature), bio02 (mean diurnal range), bio07 (temperature annual range), bio08 (mean temperature of wettest quarter), bio12 (annual precipitation), bio15 (precipitation seasonality) and bio17 (precipitation of driest quarter). Arrows represent the contribution degree of each bioclimatic variable.

The PCA analysis with the selected bioclimatic variables captured 73% of the total variance in the first two Principal Components (PC1: 52% and PC2: 21%; **Figure 2**). Positive values on PC1 represent areas characterized by cold and humid conditions with abundant precipitation throughout the year, lacking any temperature or precipitation seasonality. However, negative values on PC1 represent warmer and drier areas that can either have precipitation seasonality (when coupled with positive values in the PC2) or high temperature contrast (annual and diurnal), when coupled with negative values in the PC2.

### Climatic niche diversification in *Centaurium*

**Figure 3:**
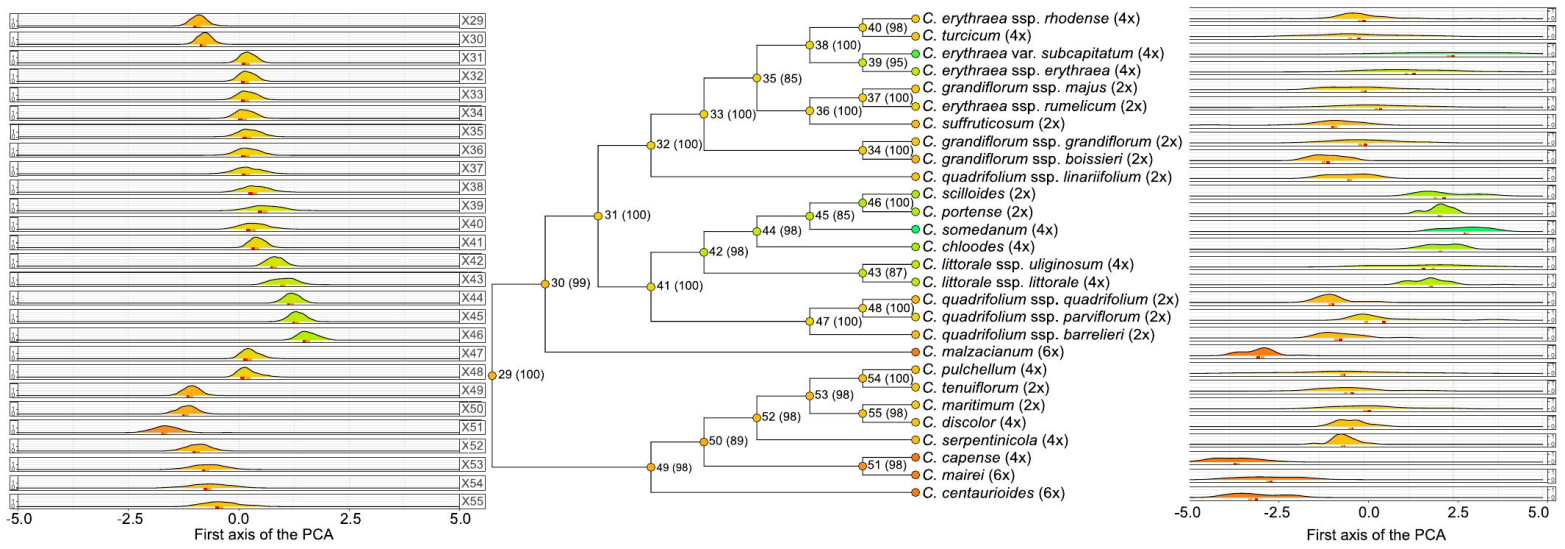
Climatic characterization of *Centaurium*. Left-side density plots represent climatic characterization of the nodes while the right-side density plots represent actual climatic characterization of the studied species. Colors in the nodes and in the tips correspond to the mean value of the PC1. Numbers at the nodes indicate node numbers, with bootstrap support in parentheses. The ploidy level of each taxon is shown at the tips.

The ancestors of *Centaurium* displayed preferences for mild dry and warm climates (**Figure 3**). These ancestral climatic niches are characterized by milder conditions (i.e., values between -1 and 1 in the PC1), whereas the current climatic niche of the taxa encompasses more extreme conditions (i.e., values up to -5 and 4.5 in the PC1). The two ancestors with the most extreme niches are that of node 51 that display a warmer niche (with values around -2.5 in the PC1), and that of node 46 that displays the coldest niche of ancestors (with values around 2.5 in the PC1).

The most recent common ancestor of *Centaurium* originated in warm areas (node 29, **Figure 3**). The subsequent nodes of the phylogeny follow different trends. On the one hand, the clade from node 30 onwards (**Figure 3**) evolved towards both mild and colder climates, except for the current niche of *C. malzacianum*. On the other hand, the clade from node 49 onwards evolved towards warmer and drier climates. Climatic niche evolution described a phylogenetic pattern, with shifts to current cold niches occurring along descendants of the cold early ancestor (node 30), whereas shifts to current warm niches occurred in the clade of the warm early ancestor (node 49). The cold early ancestor (node 30, **Figure 3**) evolved towards a clade with 19 species with cold and humid niches (node 31, **Figure 3**), as well as one species, *C. malzacianum*, that evolved towards a warm and dry climate. From the clade of node 31 (**Figure 3**), the genus splits into two clades (nodes 32 and 41, **Figure 3**). From the clade of node 32 (**Figure 3**), *Centaurium* mostly evolved towards a niche with warm and dry conditions, as it only evolved towards a cold and humid climate niche for *C. erythraea* subsp. *erythraea* and *C. erythraea* var. *subcapitatum*. In contrast, from the clade of node 41 (**Figure 3**), it evolved towards both cold and humid as well as warmer and drier niches. Lineages within the clade of node 42 (**Figure 3**) evolved towards a humid and cold climate niche: *C. scilloides*, *C. portense*, *C. somedanum*, *C. chloodes*, as well as the two subspecies of *C. littorale* (*C. littorale* subsp. *littorale* and *C. littorale* subsp. *uliginosum*) are contained in this clade. The evolution towards a warm and dry climatic niche mostly occurred for lineages within the clade of node 49 (**Figure 3**), as well as for *C. malzacianum*, as mentioned before.

### Evolution of climatic niche regarding polyploidy, life cycle and morphological traits

The evolution of climatic niche towards a single selective optimum (i.e., the null hypothesis) was not supported (**Table 1**). The best supported model was that with multiple selective optima for life cycle (**Table 1**), with the climatic niche optimum of annual/biennial being related to warmer and drier climates and the climatic optimum of perennial related to colder and more humid climates. Nevertheless, ploidy level (with two and three states) and reproductive traits (floral size, floral display and herkogamy) were also significantly supported when compared to the null hypothesis of a single optimum. The climatic niche optimum for diploids was associated with mild climates, whereas tetra- and hexaploids were related to harsh climates, with tetraploids inhabiting colder and more humid climates than hexaploids. *Centaurium* species with small flowers and high floral display had climatic niche optima in warmer and drier conditions, while species with larger flowers and low floral display were associated with colder and more humid conditions. The climatic niche optimum for high herkogamy was associated with milder conditions, while the optimum for low herkogamy was linked to more extreme conditions, including higher humidity and lower temperatures (**Table 1**). The ancestral state reconstruction for each studied trait under stochastic mapping is shown in **Supplementary Figure S3**.

**Table 1:** Results of the analyses of climatic niche evolution towards selective optima, regarding polyploidy, floral traits, and life cycle.

	*H0*	*ploidy*	*ploidybin*	*FS*	*FD*	*Hk*	*LC*
*AIC*	396.67	385.97	383.78	382.30	383.18	382.44	**380.52**
*theta*	28.11	diploid = 8.69E+01 tetraploid = -3.21E+09 hexaploid = -2.52E+09	diploid = 5.21E+09 polyploid= -9.89E+01	small/medium = -4.48E+02 large = 1.22E+10	low = 8.93E+01 high = -1.01E+10	low = 7.97E+09 high = -1.79E+02	annual/biannual = -5.45E+01 perennial = 2.20E+09
*sigma2*		1.19E+05	1.21E+05	1.12E+05	1.15E+05	1.12E+05	1.05E+05
*alpha*		1.58E-07	7.84E-08	6.60E-08	8.25E-08	1.73E-07	4.46E-07

AIC corresponds to the Akaike’s information criterion, *theta* to the optima of each model, corresponding to values of the PC1, *sigma squared* corresponds to the variance of the models, and *alpha* shows the tendency to return towards the optimum. *H0* is the null hypothesis, ploidy corresponds to the model tested with the three ploidy levels in the genus, *ploidybin* represents the model testing ploidy as binary traits (diploid vs. polyploid), *FS* represents floral size, *FD* is floral display, *Hk* shows herkogamy and *LC*, life cycle. The best fitting model for each studied trait is in bold font, considering the Akaike’s information criterion (AIC).

### Clado- and anagenetic chromosome number evolution in *Centaurium*

**Figure 4:**
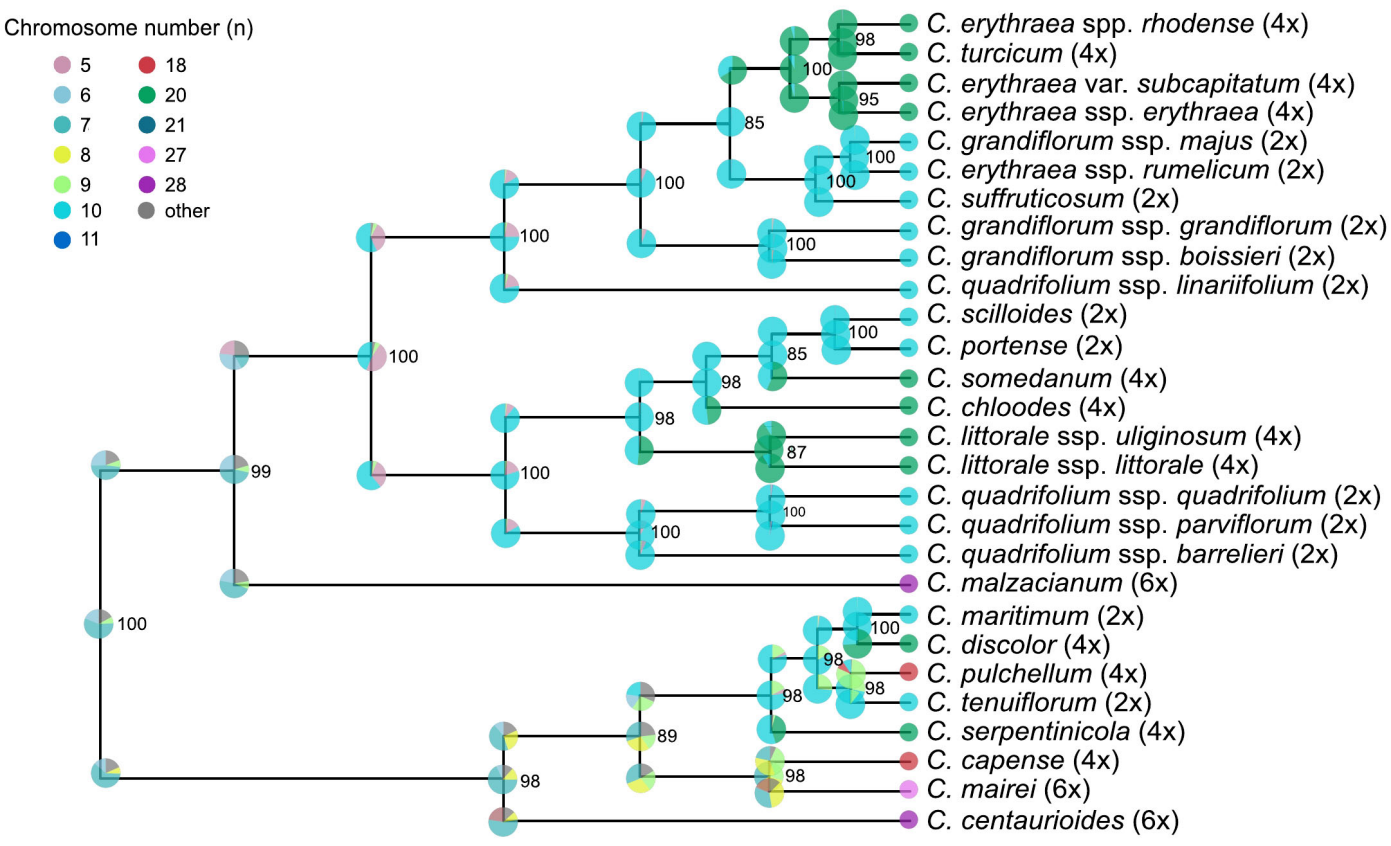
Chromosome number reconstruction based on the ChromoSSE model for the dataset. Chromosome numbers are shown with different colors and posterior probabilities with the size of the dots. Bootstrap support is displayed at the nodes.

**Figure 5:**
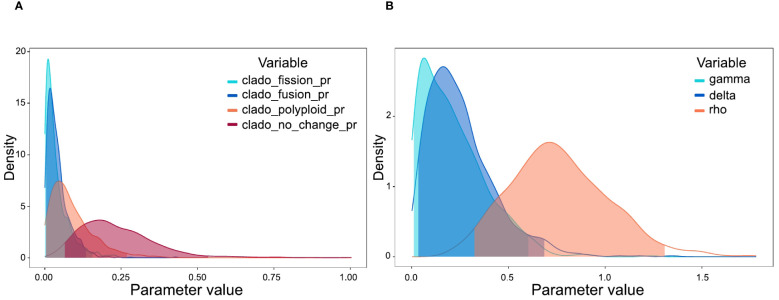
**(A)** Posterior probability densities of the estimated cladogenetic parameters in ChromoSSE model, implemented in RevBayes. The x-axis displays the rate of cladogenetic parameters, while the y-axis indicates the posterior probability density of each value. **(B)** Posterior probability densities of the estimated anagenetic parameters in ChromoSSE model, implemented in RevBayes. The x-axis displays the rate of anagenetic parameters, while the y-axis indicates the posterior probability density of each value.

Chromosome evolution analyses with ChromoSSE model showed transitions in chromosome number along the *Centaurium* phylogeny (**Figure 4**). The most likely ancestral chromosome number for the genus was *x* = 7. The posterior distribution of the cladogenetic parameters show no_change to be the most important event, followed by polyploidy cladogenetic events (**Figure 5A**). The posterior distribution of the anagenetic parameters show polyploidy to be the most important event, followed by descending dysploidy events (**Figure 5B**). Consequently, cladogenetic changes are mostly polyploidization events, while the anagenetic ones are dysploidization and polyploidization ones. Polyploid speciation was inferred in both ancestral and recent nodes that lead to terminal branches, while dysploidy events mostly occur in the terminal branches. Polyploid speciation (from n = 10 to n = 20) was inferred in the cladogenesis of *C. erythraea* subsp. *rhodense*, *C. turcicum*, *C. erythraea* var. *subcapitatum* and *C. erythraea* subsp. *erythraea*, in *C. somendanum*, *C. chloodes*, the subclade of *C. littorale* subsp. *uliginosum* and *C. littorale* subsp. *littorale*, *C. tenuiflorum, C. discolor* and C*. serpentinicola.* Besides, anagenetic chromosome transitions with dysploidy (mostly descending) were inferred for *C*. *malzacianum, C. pulchellum, C. capense, C. mairei* and *C. centaurioides*.

### Dependent vs. independent model of trait evolution and chromosome number

The results of the dependent and independent models of each of the floral traits and life cycle traits and chromosome evolution are shown in **Table 2** and in **Supplementary Table S2**. The dependent evolution of floral size, floral display and herkogamy and chromosome number evolution is better supported than the model of independent evolution of these traits. In the models studying herkogamy and floral display, the polyploidization rates in the state “2” (high herkogamy and high floral display) were significantly higher than those in state “1” (low herkogamy and low floral display). This pattern also occurred in the model studying floral size, although the difference between rates of polyploidization was not that significant. On the contrary, the model of independent evolution of life cycle and chromosome number variation was better supported, according to the AIC.

**Table 2:** Models of dependent and independent chromosome number changes correlated with floral size (*FS*), floral display (*FD*), herkogamy (*Hk*) and life cycle (*LC*).

Model	AIC
**dependent FS**	**1.40E+02**
independent FS	1.45E+02
**dependent FD**	**1.30E+02**
independent FD	1.38E+02
**dependent Hk**	**1.37E+02**
independent Hk	1.42E+02
dependent LC	1.30E+02
**independent LC**	**1.24E+02**

The best-fitted model is in bold font, considering the Akaike’s information criterion (*AIC*).

## Discussion

### Climatic niche evolution: transitions from warm and mild climates of diploids and tetraploids to harsh climates of hexaploids

Our results show the first reconstruction of the climatic niche of *Centaurium*. The most recent common ancestor of *Centaurium* originated in a mild warm and dry climate (**Figure 3**). According to Jiménez-Lobato et al. (2019) and Maguilla et al. (2021), *Centaurium* originated in the Miocene (ca. 10 Mya) in the Mediterranean Basin. Our climatic reconstruction shows that *Centaurium* evolved from an ancestral warm and dry climate niche, which can be associated with the Mediterranean Basin (Jiménez-Lobato et al., 2019; Maguilla et al., 2021), to both colder and warmer climate niches (**Figure 3**). Besides, the colder and humid climate niches showed a remarkable variation, as some *Centaurium* species thrive in coastal areas (i.e., the tetraploid *C. littorale* subsp. *littorale*, the tetraploid *C. chloodes* or the diploid *C. scilloides*), and others in inland regions (i.e., the tetraploids *C. portense* and *C. somedanum*; see **Supplementary Figure S2**) (Díaz-Lifante, 2012). These two regions hold remarkable climatic differences: coastal areas display relatively stable and moderate temperature fluctuations throughout the year, as well as a constant high level of humidity. In contrast, inland regions often experience more significant temperature fluctuations. However, despite the large climatic differences between these areas, *Centaurium* species tend to occupy the same niches.

The climatic niche characterization of the genus in its phylogeny shows an interesting pattern: clades with milder ancestral niches (e.g. nodes 32 and 42) exhibit greater species richness compared to those transitioning into warmer and drier ones (e.g., from node 49 onwards) (**Figure 3**). This pattern may be favored by the climatic niche of the ancestors, as they remain in a mild climate, promoting shifts towards warmer or colder ones. This maintenance of the ancestors’ mild climate invokes niche conservatism, at least in the ancestor’s climatic niche. Niche conservatism is the tendency of a species to retain its climatic requirements across time (Peterson et al., 1999), and has been proposed to facilitate allopatric speciation (Wiens, 2004). Considering the sympatric origin of the species due to hybridization events (Zeltner, 1970; Mansion et al., 2005; Jiménez-Lobato et al., 2019; Maguilla et al., 2021; Valdés-Florido et al., 2024) with no geographical barrier to get isolated, and the climatic niche conservatism reported here, *Centaurium* could represent an example of climatic niche conservatism playing an important role for sympatric speciation. However, we should consider that our climate data are geographically very coarse, whereas the differentiation necessary for speciation may happen at a much finer resolution. The importance of niche conservatism in sympatric speciation was suggested by Wiens (2004). Besides, climatic niche breadth changes along the evolutionary history of the genus. In the reconstructed ancestral climatic niche, climatic niches from the species of node 30 onwards are broader than those of the node 49 onwards (**Figure 3**). Climatic niches reconstructed in the nodes are narrower than the niches reconstructed at the tips (**Figure 3**). The species that have retained the ancestral niche have mildly expanded it (e.g., the tetraploid *C. erythraea* subsp. *rhodense* and the diploid *C. quadrifolium* subsp. *quadrifolium*), whereas others have undergone a complete niche shift (e.g., the hexaploids *C. malzacianum* and *C. mairei*). Notably, only the two subspecies of *C. erythraea* have marginally broadened their niche, yet persisting within the intermediate zones as part of their niche. Conversely, the hexaploids (i.e., *C. malzacianum, C. mairei*, *C. centaurioides*) and one tetraploid (*C. capense*) have undergone a radical shift to niches characterized by warmer, drier, and more disparate conditions, distinct from those of any other species. Additionally, the niche of taxa as *C. scilloides* has evolved towards niches characterized by wetter, colder, and less disparate environments. As mentioned, some *Centaurium* species have originated by hybridization. For example, the hexaploids and *C. capense* have been suggested to be allopolyploids (Valdés-Florido et al., 2024), so that there has not been any geological barrier involved in the speciation process. Thus, the pattern of niche broadening could be beneficial to reduce niche competitiveness, and then enhance the likelihood of the lineage to survive (Carscadden et al., 2020).

### Interplay between polyploid evolution, morphological traits, and climatic niche

Polyploidy, both allo- and autopolyploidy, is a key event in the evolution of *Centaurium* (Zeltner, 1970; Mansion et al., 2005; Jiménez-Lobato et al., 2019; Maguilla et al., 2021; Valdés-Florido et al., 2024). In fact, the climatic niche of *Centaurium* evolves towards selective optima dictated by ploidy levels. In this genus, polyploidization has been proposed to have occurred due to hybridization events (allopolyploidy) or as result of autopolyploidy (Zeltner, 1970; Mansion et al., 2005; Jiménez-Lobato et al., 2019; Maguilla et al., 2021; Valdés-Florido et al., 2024). Thus, polyploidy in *Centaurium* is significantly linked to hybridization as well as to climatic niche evolution, the latter evolving towards ploidy level optima (**Table 1**). Climatic niches associated with mild conditions are related to diploids, while harsh ones to tetra- and hexaploids (**Table 1**), with tetraploids inhabiting colder and more humid areas than hexaploids. Regarding life cycle, our results supported that climatic niche evolves towards life cycle optima. The climatic niche optimum of annual/biennial is related to warmer and drier climates and the climatic optimum of perennial is related to colder and more humid climates (**Table 1**). The related evolution of life cycle considering climate has also been confirmed in *Centaurium* (Jiménez-Lobato et al., 2019). Four transitions from perennial life cycle to annual/biennial were inferred: one between 10.25 Mya and 7.29 Mya, two in the Pleistocene (one around 0.7 Mya and the other around 1.72 Mya), and the last transition with uncertain timing (Jiménez-Lobato et al., 2019). Some of these transitions to annual/biennial life forms are associated with climatic changes in the Mediterranean, as the arid period occurred around the Messinian Salinity Crisis (MSC) around 5.96 - 5.33 Mya or the seasonal drought of the Mediterranean climate (3.4 - 2.8 Mya).

In flowering plants, selection on floral traits and plant mating strategies leading to a system in which seed production is ensured (i.e., reproductive assurance), is key to guarantee the maintenance of the lineage (Lloyd and Schoen, 1992). The climatic niche evolution towards different herkogamy optima was also supported, as well as the models of the other studied traits (i.e., floral display and floral size). The climatic niche optimum for high herkogamy was associated with milder conditions and the climatic optimum of low herkogamy was related to colder and more humid conditions (**Table 1**). Species with small/medium flowers and with high floral display are related to warmer and drier conditions, and those with larger flowers and low floral display, to colder and humid ones (**Table 1**). These floral traits are related to selfing syndrome, modifying the attraction to pollinators, or the spatial distance between the anthers and stigma, which facilitate selfing (Lloyd and Schoen, 1992; Goodwillie et al., 2010). For example, in *C. erythraea* it has been reported that changes in floral size and floral display as well as in the levels of herkogamy are related to the quantity of pollinators in the environment. In pollinator-poor environments there are fewer and smaller flowers of this species with lower levels of herkogamy compared to pollinator-rich environments, conferring reproductive assurance via selfing (Brys and Jacquemyn, 2011). Thus, the evolution of floral traits is dependent on climatic niche as well as on the pollinator’s richness, which ultimately depends on climate (Rech et al., 2016).

### Chromosome evolution, reproductive traits, and life cycle

The results of the ChromoSSE analysis suggest a most probable base chromosome number of *x* = 7 (**Figure 4**). However, this result is questionable, as the ChromoSSE model does not include the possibility of base-number reconstruction parameter in the analyses, contrary to other approaches such as ChromEvol (Glick and Mayrose, 2014). Despite *x* = 7 being the most probable base chromosome number, chromosome numbers changed throughout the evolutionary history of the genus, ultimately stabilizing at *x* = 10 (**Figure 4**). This base chromosome number of *x* = 10 was also suggested by previous studies (Zeltner, 1970; Maguilla et al., 2021; Escudero et al., 2023). Polyploidy has been identified as the most important anagenetic event, as well as the second most important cladogenetic event, with that of no_change being the most important cladogenetic event. This estimation is congruent with all the previous studies of the genus that suggest the crucial role of polyploidy in *Centaurium* diversification (Zeltner, 1970; Mansion and Struwe, 2004; Mansion et al., 2005; Guggisberg et al., 2006; Escudero et al., 2023; Valdés-Florido et al., 2024). Whereas most of the transition events from diploid to tetraploid are linked to transitions from drier and warmer to colder and wetter climatic niches and from southern to northern ranges (considering the distribution of the genus, **Figure 1**), transitions leading to the hexaploids *C. malzacianum*, *C. centaurioides* and *C. mairei*, and the tetraploid *C. capense*, coincide with the transition from temperate to warmer and drier climatic niche in the southern limit of the genus range (Maguilla et al., 2021).

The global distribution pattern of polyploids reveals a latitudinal trend, so that polyploid frequency increases with increasing latitude, i.e., areas of cold climates (Stebbins, 1971; Rice et al., 2019). Several studies (Levin, 2019; Lavania, 2020; Vimala et al., 2021) support autopolyploids to have originated due to temperature stress, so that they are distributed in colder areas. However, our results suggest a different pattern with the allo-hexaploids and the allo- tetraploid *C. capense* (Valdés-Florido et al., 2024), distributed in warmer and southern areas (**Supplementary Figure S2**). This trend of polyploids inhabiting warmer areas is also observed in autopolyploids as *Solidago canadensis* L. (Cheng et al., 2021). In their experimental study, Cheng et al. (2021) demonstrated that angiosperms can also expand their distribution towards warmer regions through heat tolerance evolution given by polyploidization. Our results suggest that this pattern could occur not only in autopolyploids, but also in allopolyploid species.

Chromosome number variations (i.e., polyploidy) have been suggested to promote morphological changes (Stebbins, 1950). This association is confirmed in *Centaurium*, as the dependent evolution of chromosome number and floral size, floral display and herkogamy has been shown by our analyses (**Table 2**; **Supplementary Table S2**). In fact, some studies in *Centaurium* revealed morphological differences between diploids and tetra- and hexaploids (Valdés-Florido et al., 2024). However, Jiménez-Lobato et al. (2019) tested the dependent evolution of these traits and polyploidy using Pagel’s model, finding independent evolution for those traits. This is incongruent with our results of the dependent evolution of morphological traits and chromosome number evolution using a more complex model. In this model, the morphological traits are modeled by a Markov model for binary traits whereas chromosome evolution is modeled using a specific model for chromosome evolution (**Table 2**; **Supplementary Table S2**). Additionally, the higher rates of polyploidization estimated in *Centaurium* taxa with high herkogamy and high floral display (**Table 2**; **Supplementary Table S2**) are explained by polyploidization events in terminal short branches of the phylogeny (this model of chromosome evolution did not include chromosome evolution at cladogenesis and most of the polyploid events are inferred in the terminal branches).

Perenniality has traditionally been related to polyploidy (Stebbins, 1950; Thompson and Lumaret, 1992; Otto and Whitton, 2000; Rice et al., 2019). Polyploids typically exhibit slower growth compared to diploids due to the extended duration required for DNA replication and cell division resulting from higher chromosome numbers (Stebbins, 1950; Levin, 2002). Consequently, they are expected to be perennial rather than annual. However, our analyses suggest that life cycle has not been related to the evolution of chromosome number in *Centaurium*.

## Conclusions

Our study on the climatic niche evolution and chromosome number dynamics within the genus *Centaurium* provides valuable insights into the interplay between climatic adaptation and genomic and morphological changes. We confirm that the genus originated in a climate with mild conditions, based on the reconstruction of the climatic niche of the ancestors, which could be the Mediterranean Basin (Jiménez-Lobato et al., 2019; Maguilla et al., 2021). We also show the association between climatic niche and polyploidization events. Additionally, the presence of polyploids also in warmer regions indicates that some *Centaurium* species (tetra- and hexaploid species) do not always follow traditional latitudinal trends (Stebbins, 1971; Rice et al., 2019). The dependent evolution between floral traits and chromosome number evolution has been confirmed, as well as the independent evolution between life cycle and chromosome number evolution. This study offers valuable insights into the strategies that angiosperms (here Mediterranean lineages) employ to grow in diverse life cycles and climates as well as the importance of considering both climatic and genomic factors to understand species diversification.

## Data Availability

The original contributions presented in the study are included in the article/**Supplementary Material**, further inquiries can be directed to the corresponding author/s.
