# Supplementary material for: The interplay between climatic niche evolution, polyploidy and reproductive traits explains plant speciation in the Mediterranean Basin: a case study in Centaurium (Gentianaceae)
Source: Front Plant Sci. 2024 Aug 9;15:1439985. doi: 10.3389/fpls.2024.1439985 (PMC11344271; doi:10.3389/fpls.2024.1439985)

Figure S1. Distribution maps of each taxon within *Centaurium*.

Centaurium capense

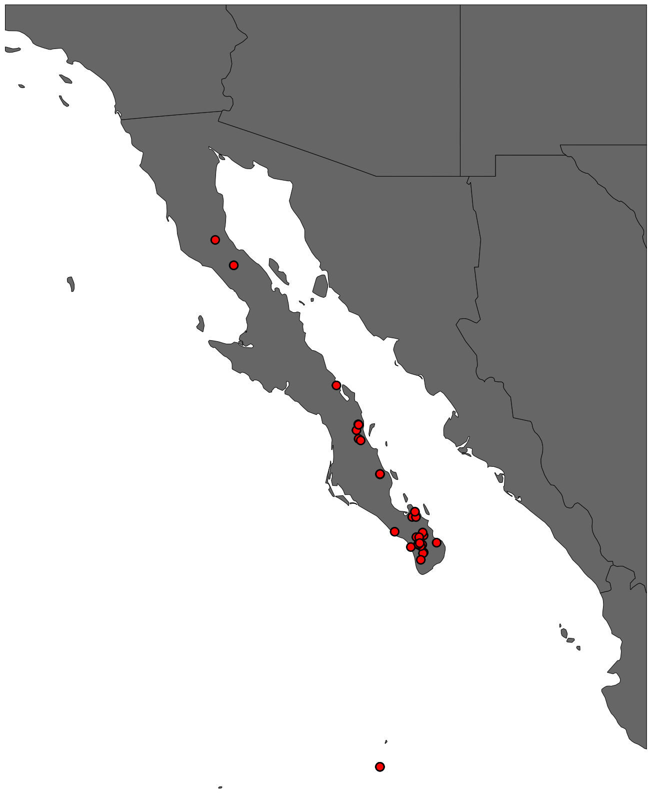

Centaurium centaurioides

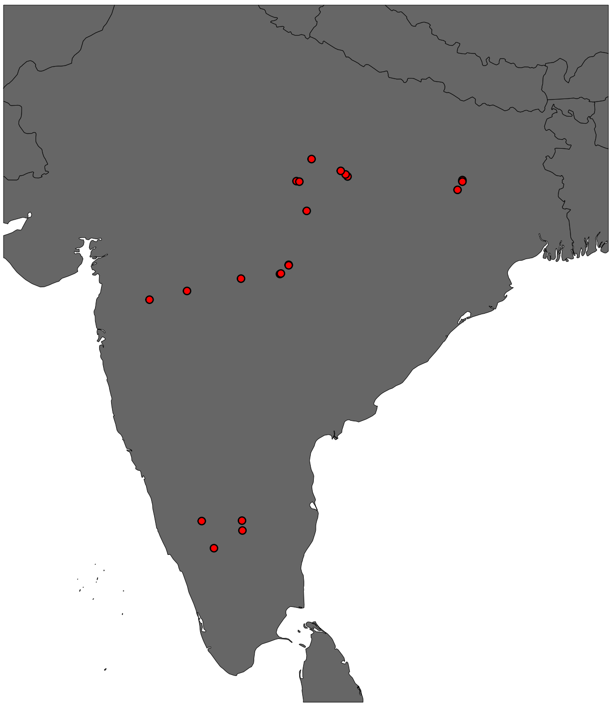

Centaurium chloodes

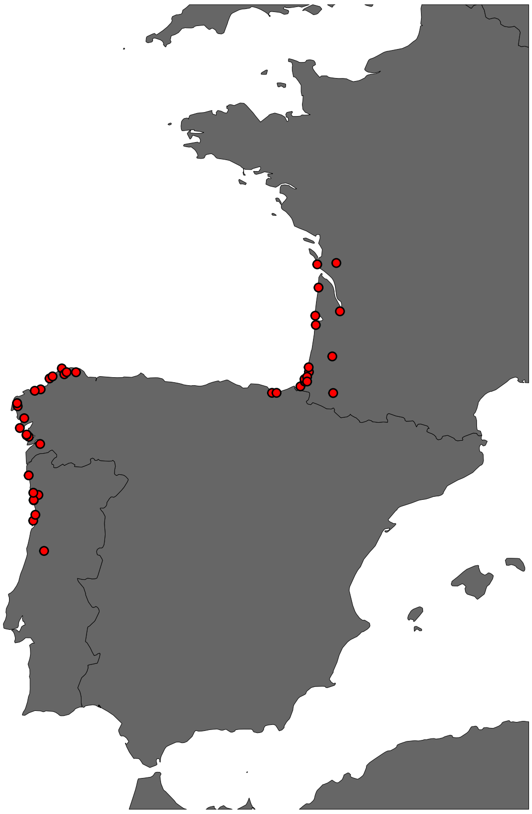

Centaurium discolor

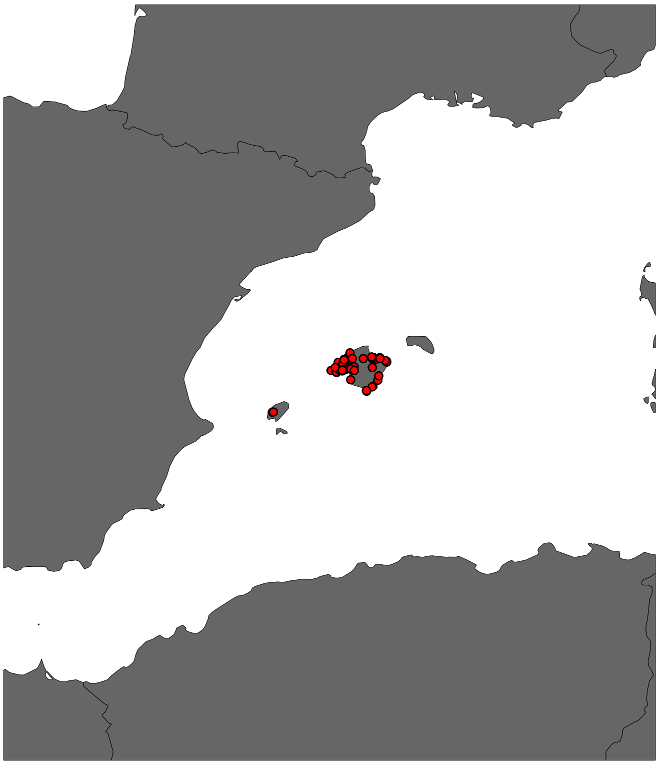

Centaurium erythraea ssp. erythraea

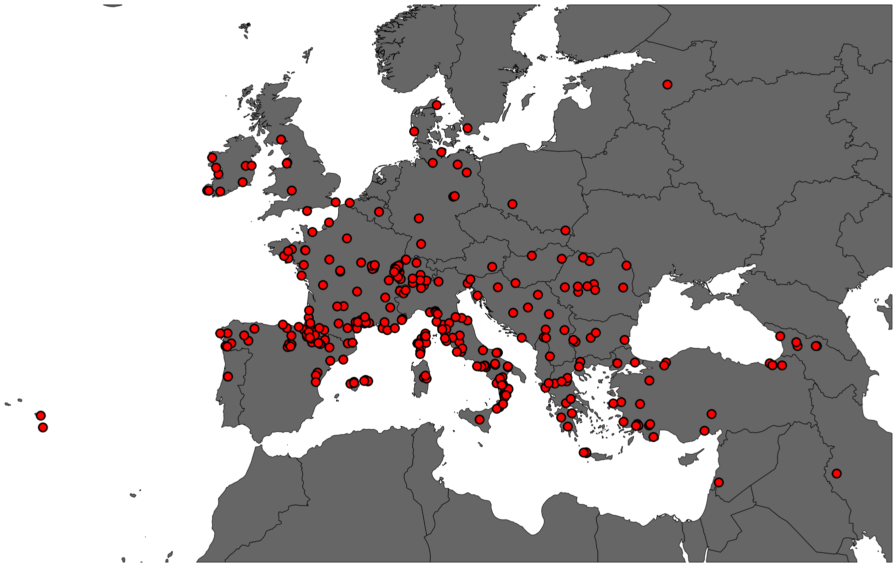

Centaurium erythraea ssp. rhodense

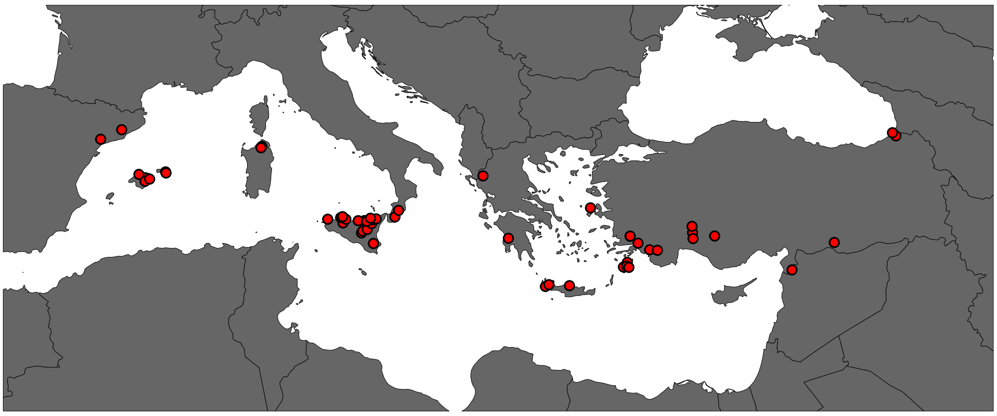

Centaurium erythraea ssp. rumelicum

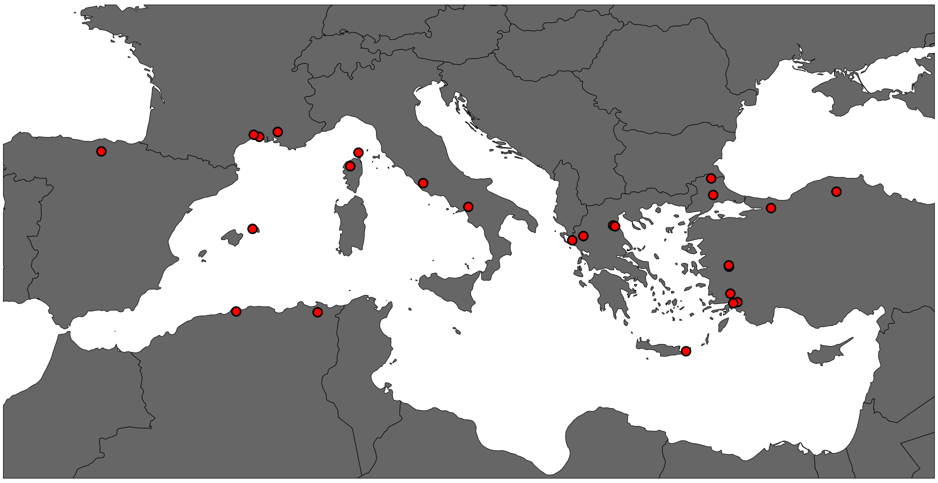

Centaurium erythraea var. subcapitatum

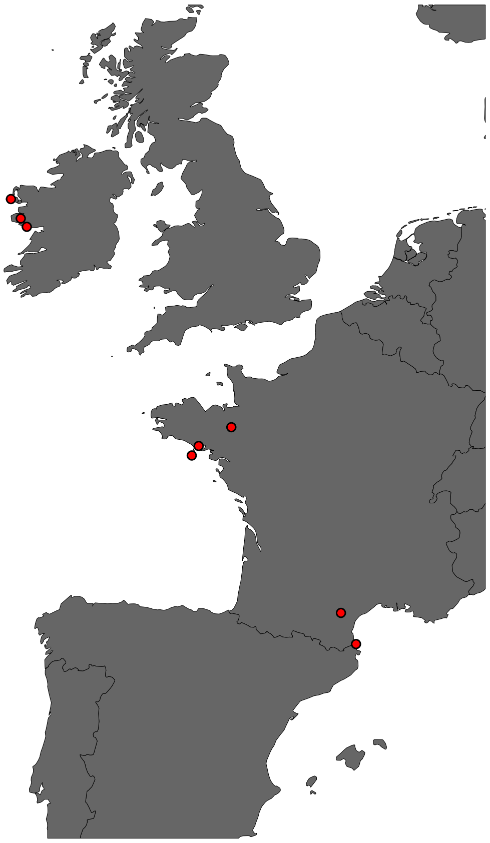

Centaurium grandiflorum ssp. boissieri

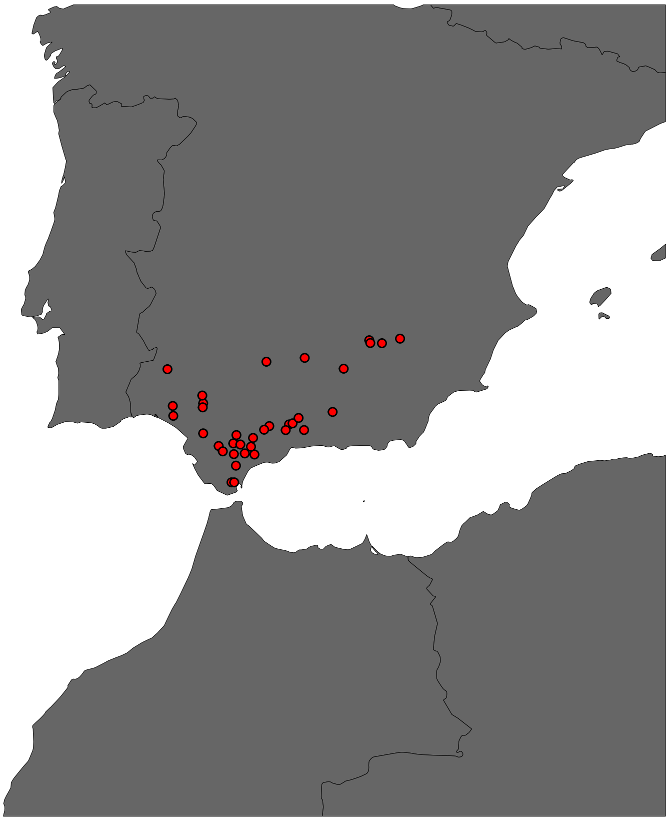

Centaurium grandiflorum ssp. grandiflorum

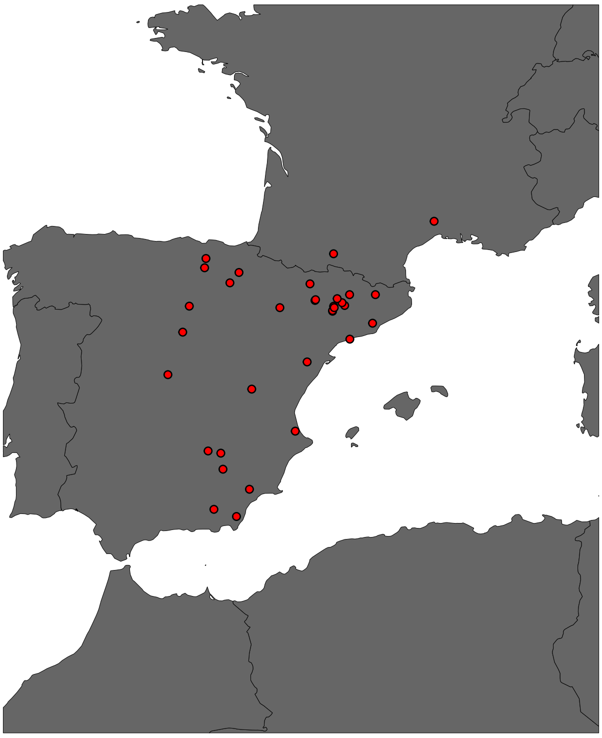

Centaurium grandiflorum ssp. majus

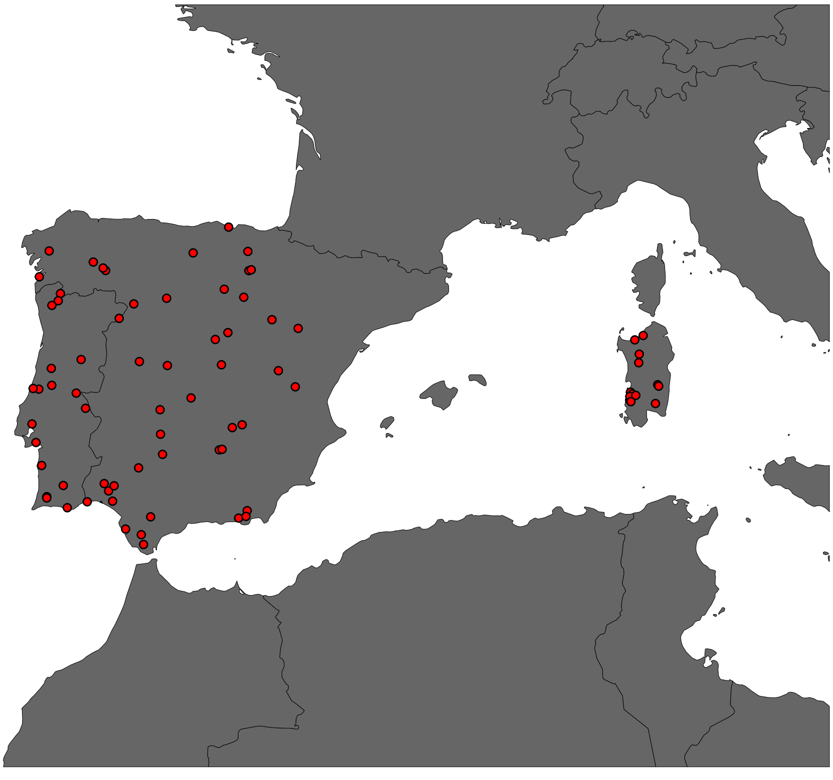

Centaurium littorale ssp. littorale

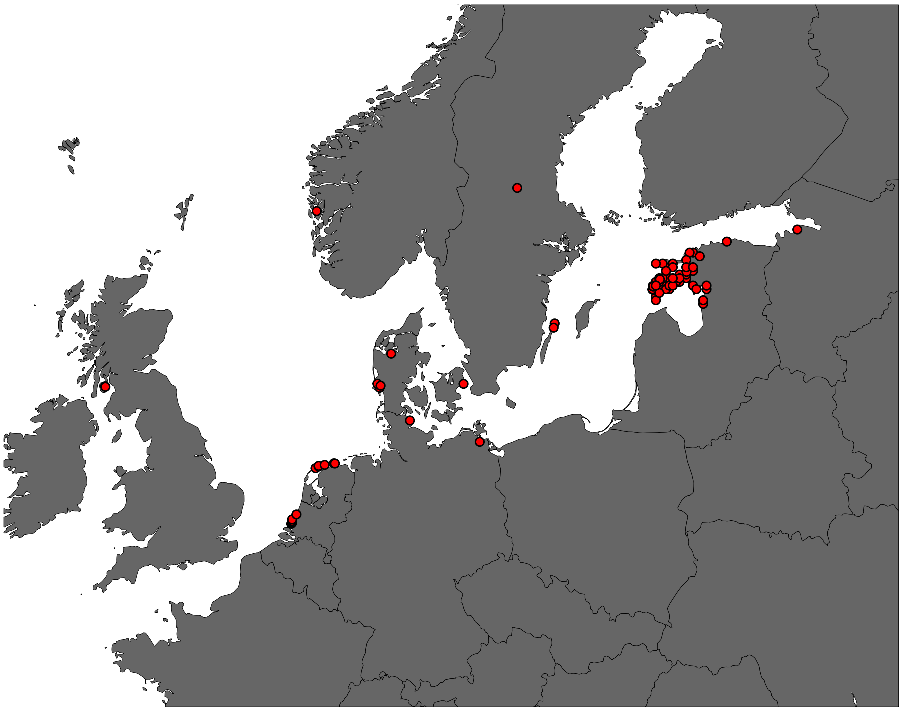

Centaurium littorale ssp. uliginosum

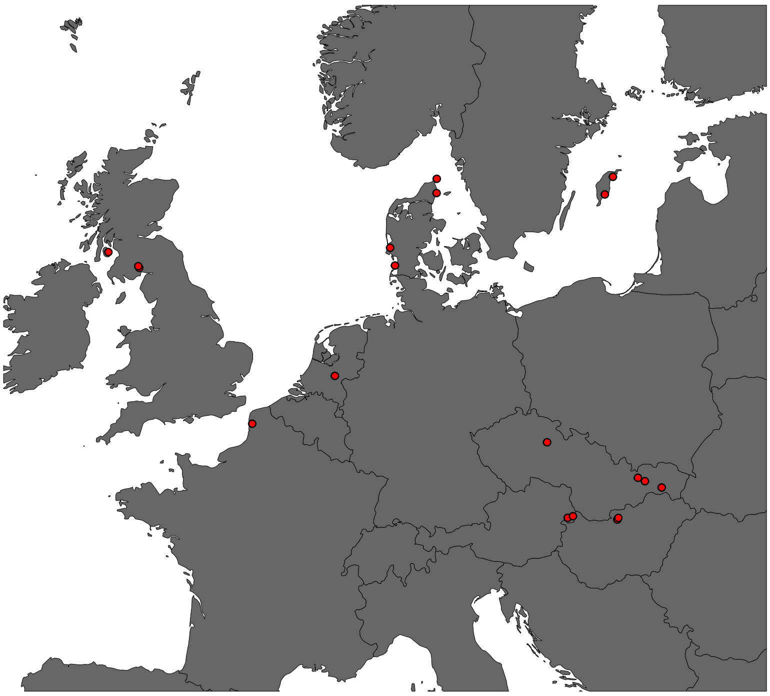

Centaurium mairei

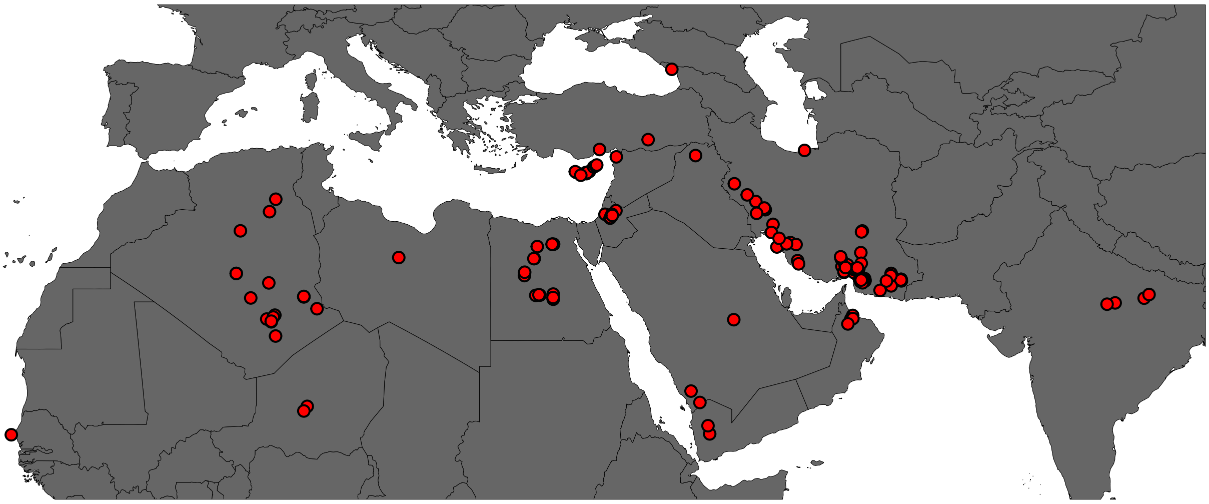

Centaurium malzacianum

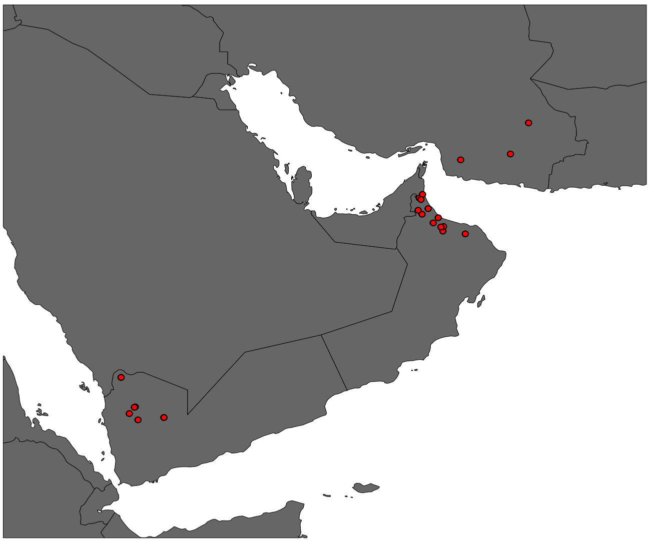

Centaurium maritimum

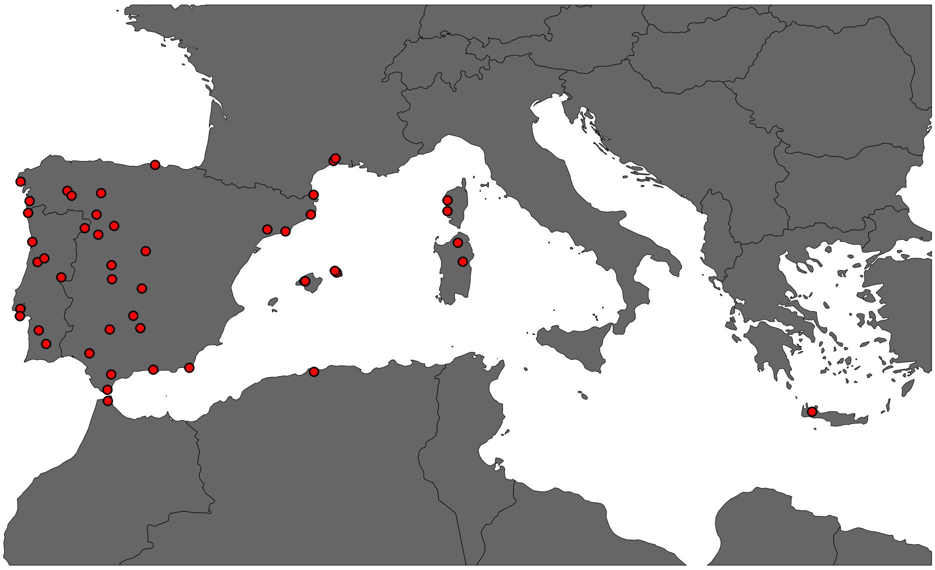

Centaurium portense

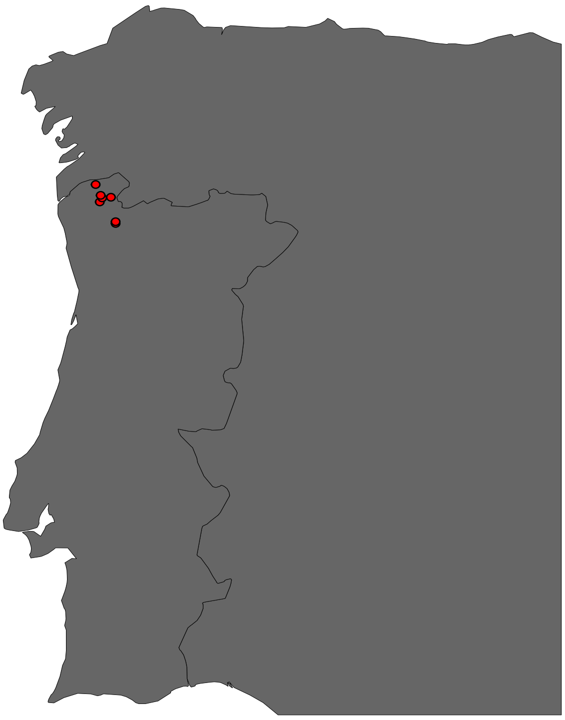

Centaurium pulchellum

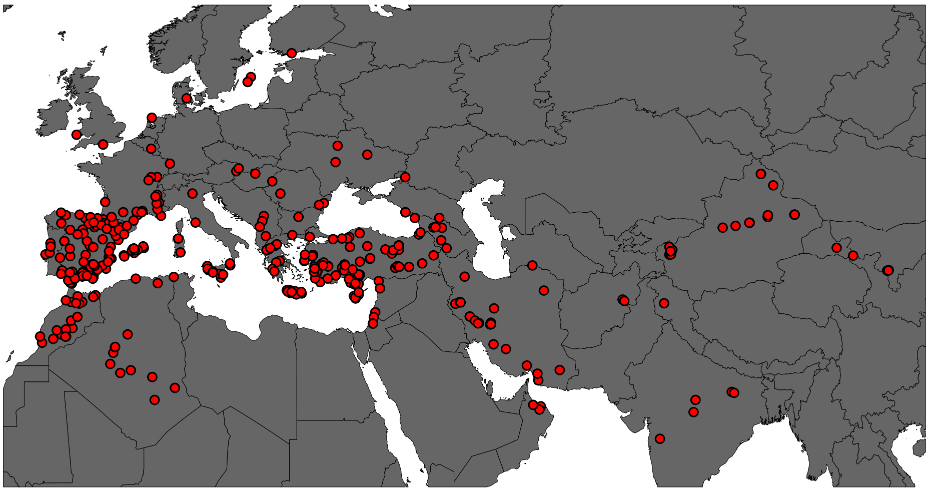

Centaurium quadrifolium ssp. barrelieri

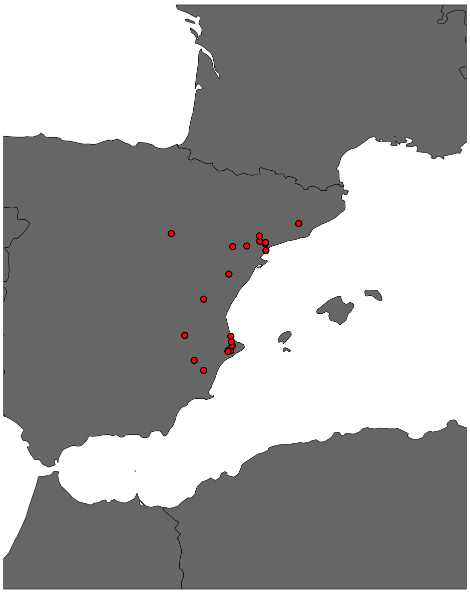

Centaurium quadrifolium ssp. linariifolium

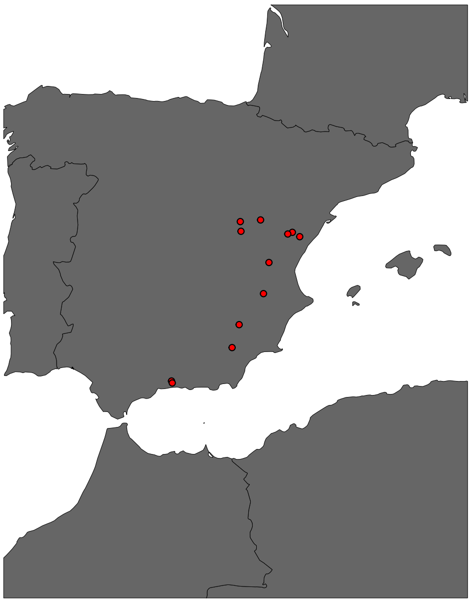

Centaurium quadrifolium ssp. parviflorum

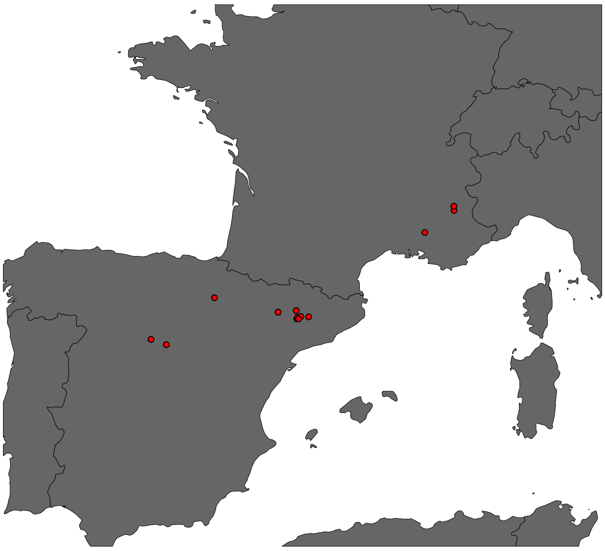

Centaurium quadrifolium ssp. quadrifolium

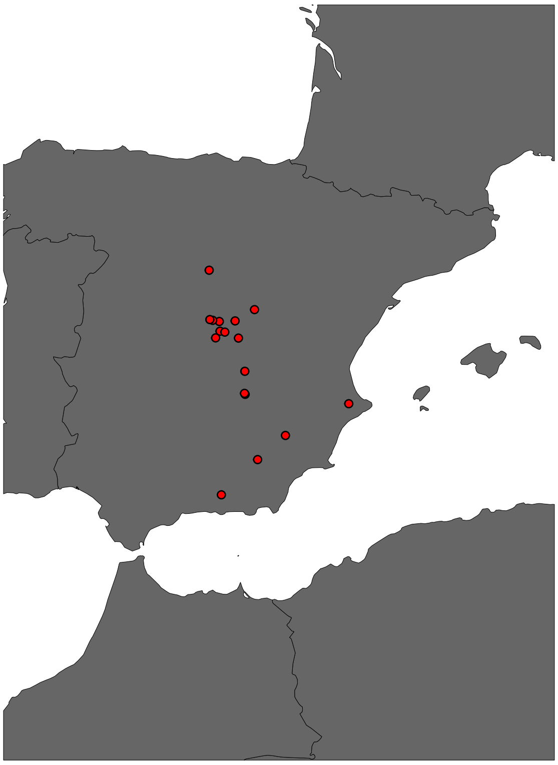

Centaurium scilloides

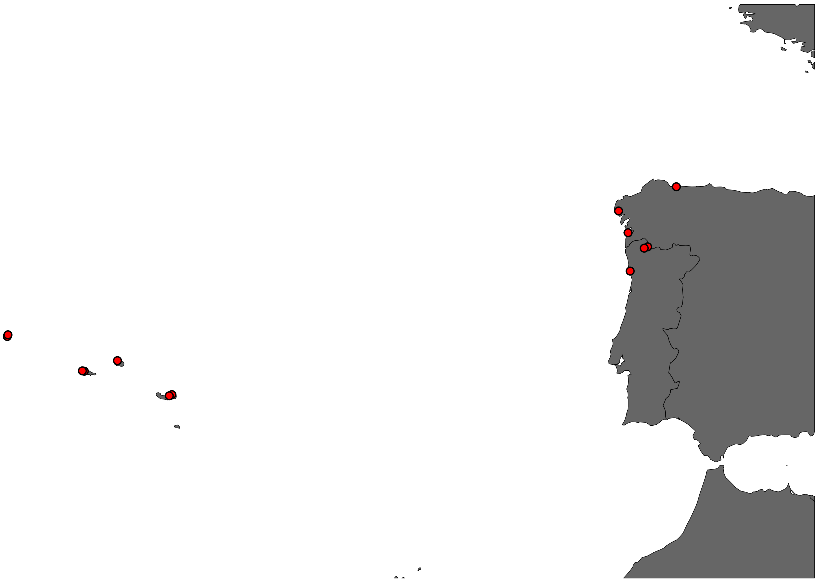

Centaurium serpentinicola

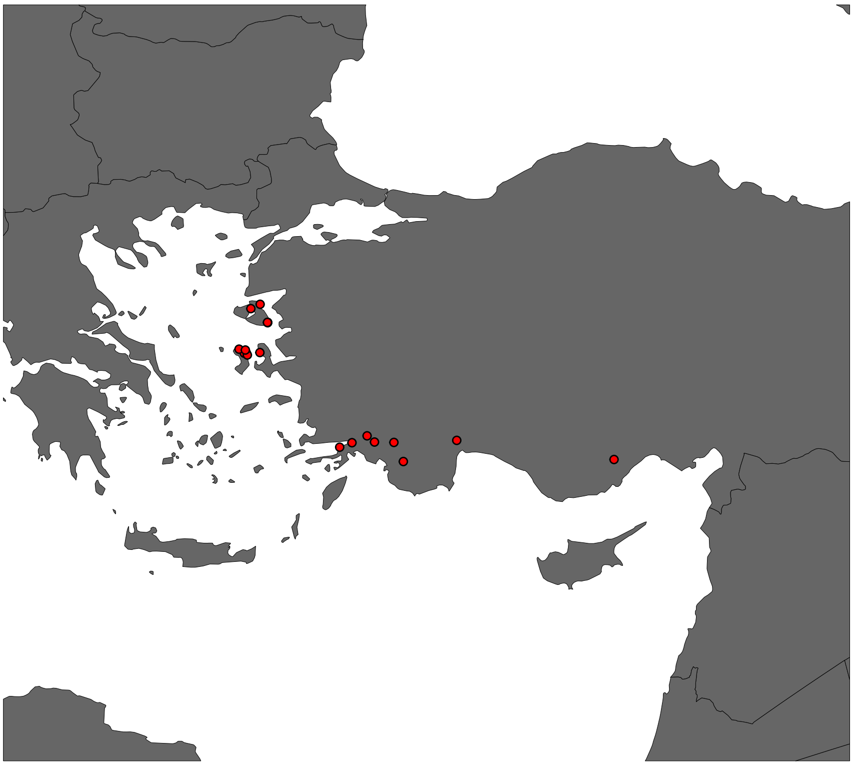

Centaurium somedanum

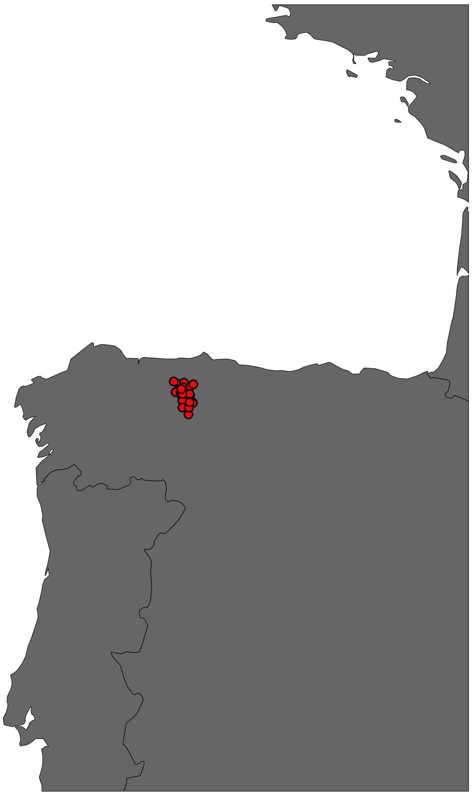

Centaurium suffruticosum

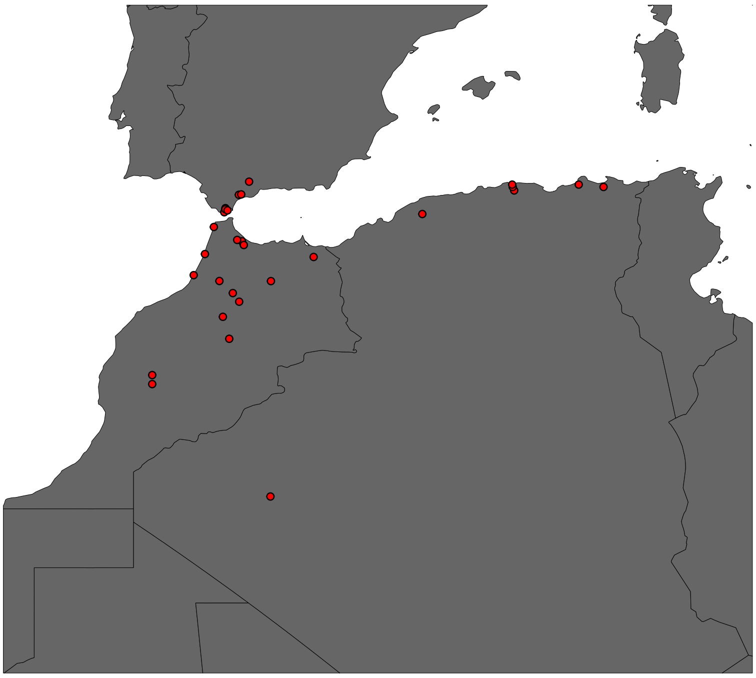

Centaurium tenuiflorum

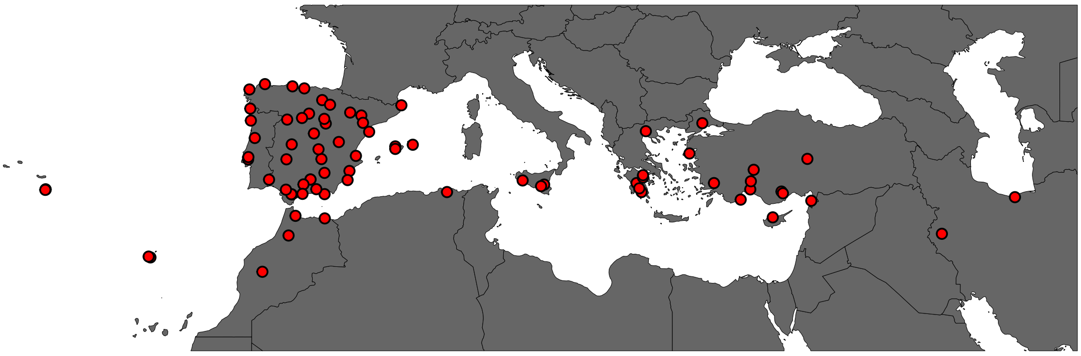

Centaurium turcicum

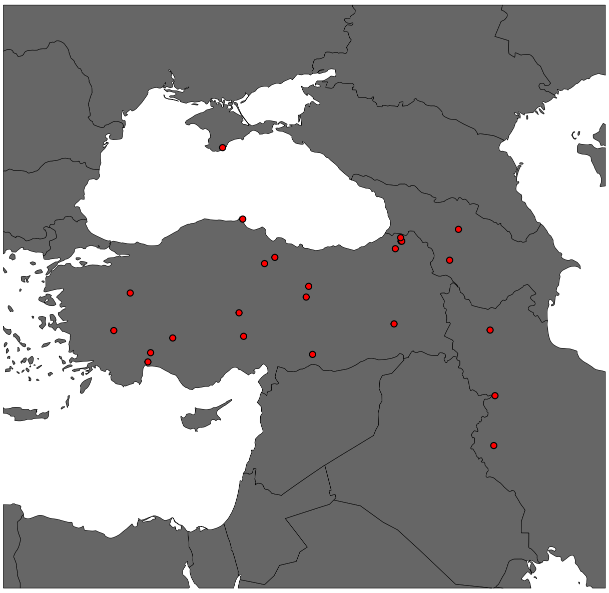

Supplement: Supplementary file 1 [file Image_1.pdf]
