# Supplementary material for: The interplay between climatic niche evolution, polyploidy and reproductive traits explains plant speciation in the Mediterranean Basin: a case study in Centaurium (Gentianaceae)
Source: Front Plant Sci. 2024 Aug 9;15:1439985. doi: 10.3389/fpls.2024.1439985 (PMC11344271; doi:10.3389/fpls.2024.1439985)

Figure S2. Dendrogram of the used bioclimatic variables. Bioclimatic variables enclosed in red were the ones used in the analyses.

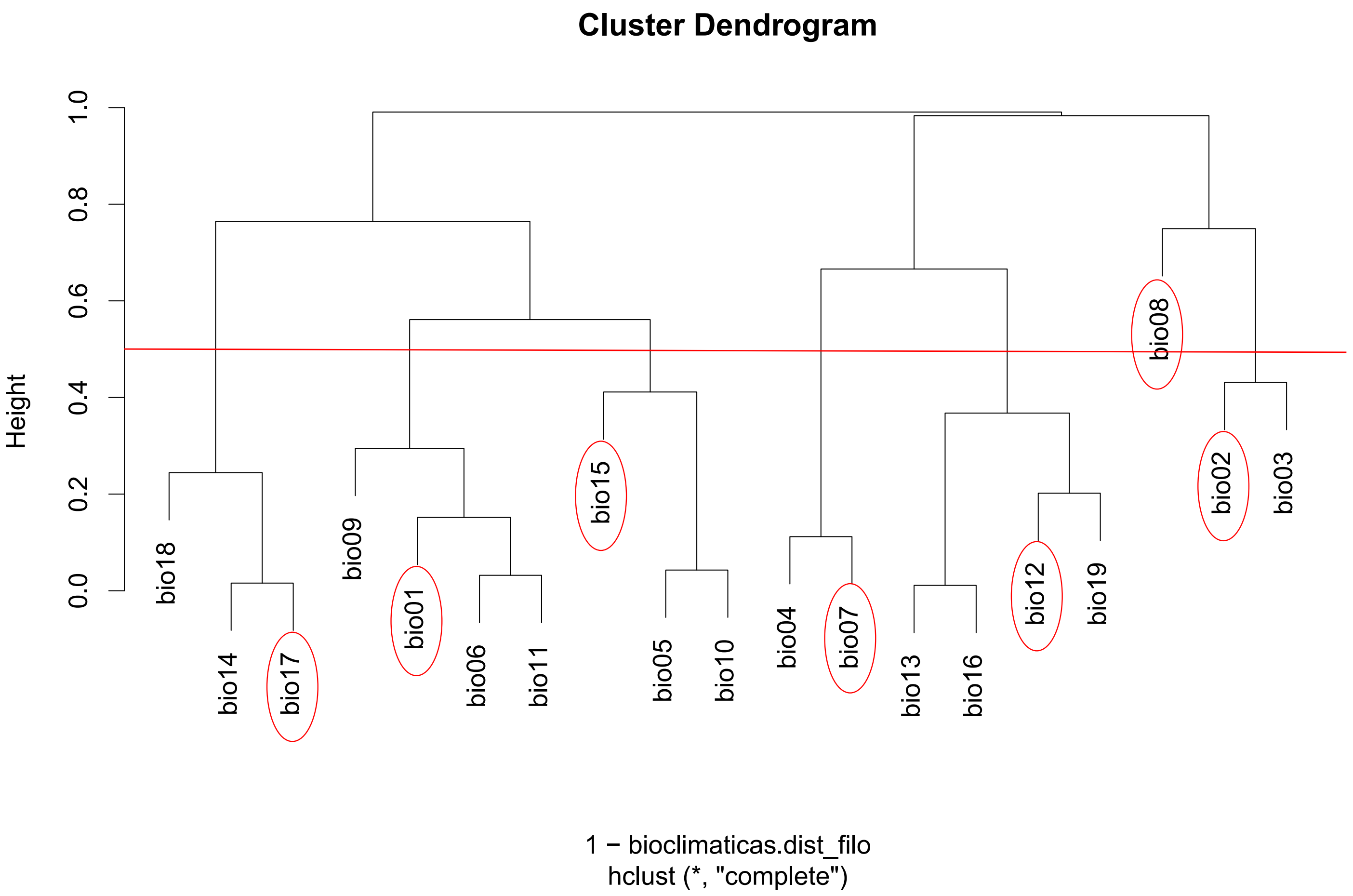

Supplement: Supplementary file 2 [file Image_2.pdf]
