# Supplementary material for: The interplay between climatic niche evolution, polyploidy and reproductive traits explains plant speciation in the Mediterranean Basin: a case study in Centaurium (Gentianaceae)
Source: Front Plant Sci. 2024 Aug 9;15:1439985. doi: 10.3389/fpls.2024.1439985 (PMC11344271; doi:10.3389/fpls.2024.1439985)
Supplement: Supplementary file 3 [file Image_3.pdf]

Figure S3. Reconstruction of ancestral states under stochastic mapping of **A)** ploidy levels (2x, 4x and 6x) **B)** ploidy level (diploid vs. polyploid), **C)** floral size (small to medium vs. large), **D)** floral display (low vs. high), **E)** herkogamy (low herkogamy vs. high-herkogamy) and **F)** life cycle (annual/biennial vs. perennial).

A)

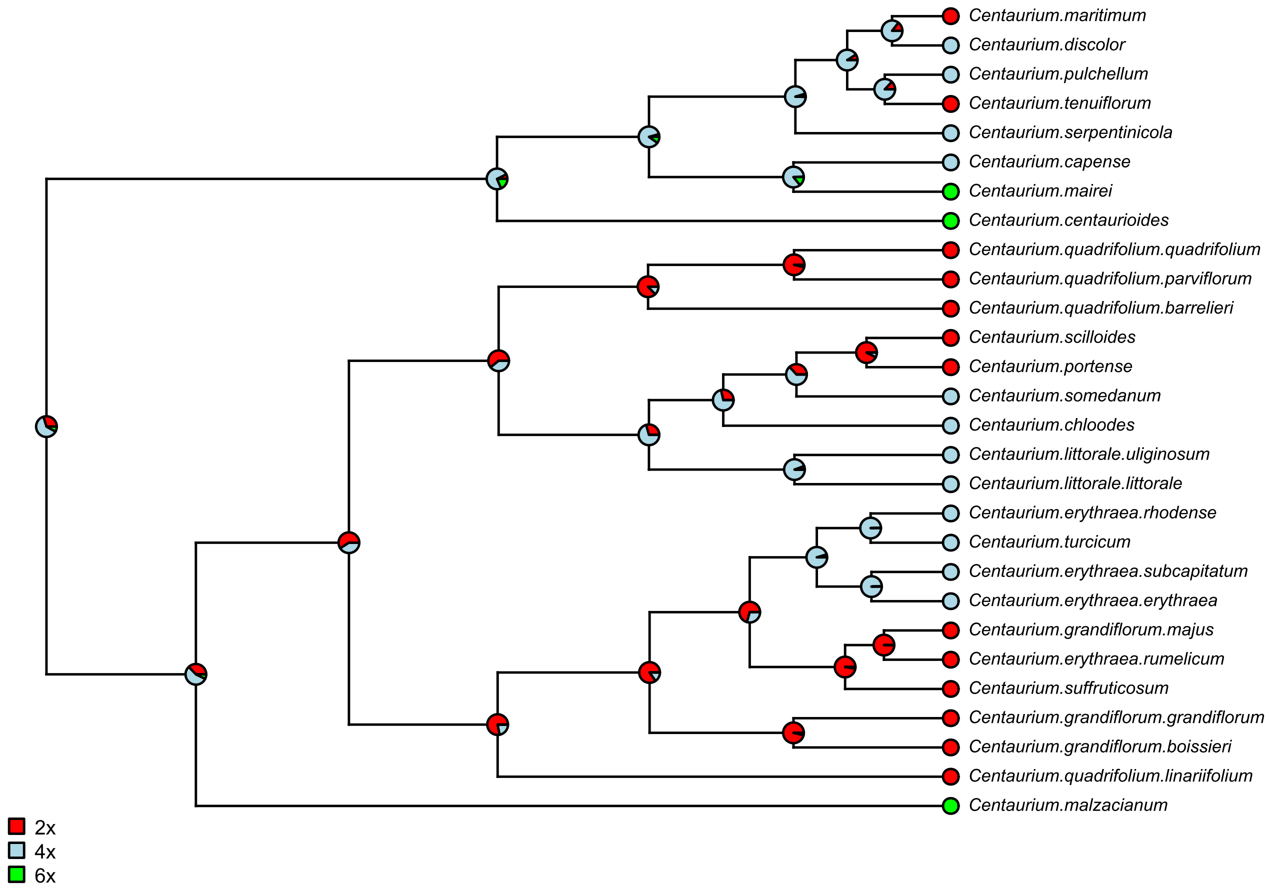

B)

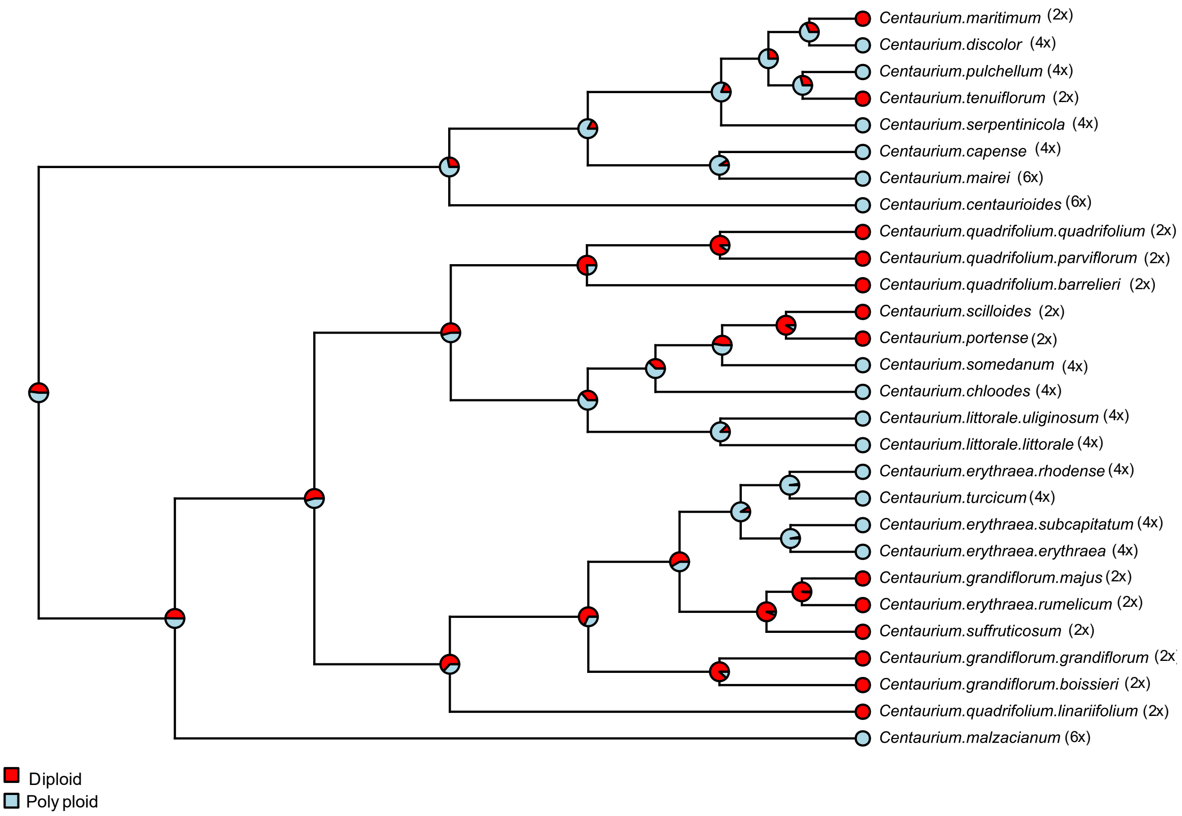
Centaurium.maritimum (2x)
Centaurium.discolor (4x)
Centaurium.pulchellum (4x)
Centaurium.tenuiflorum (2x)
Centaurium.serpentinicola (4x)
Centaurium.capense (4x)
Centaurium.mairei (6x)
Centaurium.centaurioides (6x)
Centaurium.quadrifolium.quadrifolium (2x)
Centaurium.quadrifolium.parviflorum (2x)
Centaurium.quadrifolium.barrelieri (2x)
Centaurium.scilloides (2x)
Centaurium.portense (2x)
Centaurium.somedanum (4x)
Centaurium.chloodes (4x)
Centaurium.littorale.uliginosum (4x)
Centaurium.littorale.littorale (4x)
Centaurium.erythraea.rhodense (4x)
Centaurium.turcicum (4x)
Centaurium.erythraea.subcapitatum (4x)
Centaurium.erythraea.erythraea (4x)
Centaurium.grandiflorum.majus (2x)
Centaurium.erythraea.rumelicum (2x)
Centaurium.suffruticosum (2x)
Centaurium.grandiflorum.grandiflorum (2x
Centaurium.grandiflorum.boissieri (2x)
Centaurium.quadrifolium.linariifolium (2x)
Centaurium.malzacianum (6x)
Diploid
Poly ploid

C)

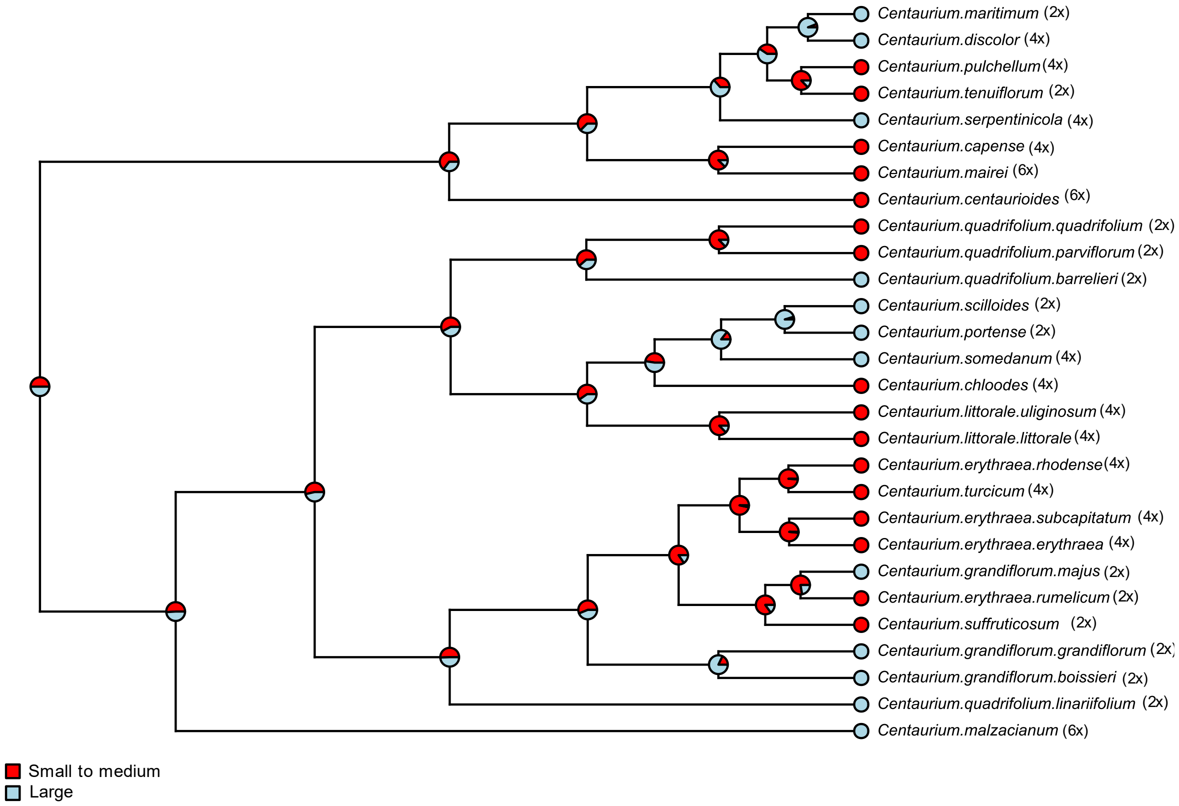
Centaurium.maritimum (2x)
Centaurium.discolor (4x)
Centaurium.pulchellum (4x)
Centaurium.tenuiflorum (2x)
Centaurium.serpentinicola (4x)
Centaurium.capense (4x)
Centaurium.mairei (6x)
Centaurium.centaurioides (6x)
Centaurium.quadrifolium.quadrifolium (2x)
Centaurium.quadrifolium.parviflorum (2x)
Centaurium.quadrifolium.barrelieri (2x)
Centaurium.scilloides (2x)
Centaurium.portense (2x)
Centaurium.somedanum (4x)
Centaurium.chloodes (4x)
Centaurium.littorale.uliginosum (4x)
Centaurium.littorale.littorale (4x)
Centaurium.erythraea.rhodense (4x)
Centaurium.turcicum (4x)
Centaurium.erythraea.subcapitatum (4x)
Centaurium.erythraea.erythraea (4x)
Centaurium.grandiflorum.majus (2x)
Centaurium.erythraea.rumelicum (2x)
Centaurium.suffruticosum (2x)
Centaurium.grandiflorum.grandiflorum (2x)
Centaurium.grandiflorum.boissieri (2x)
Centaurium.quadrifolium.linariifolium (2x)
Centaurium.malzacianum (6x)
Small to medium
Large

D)

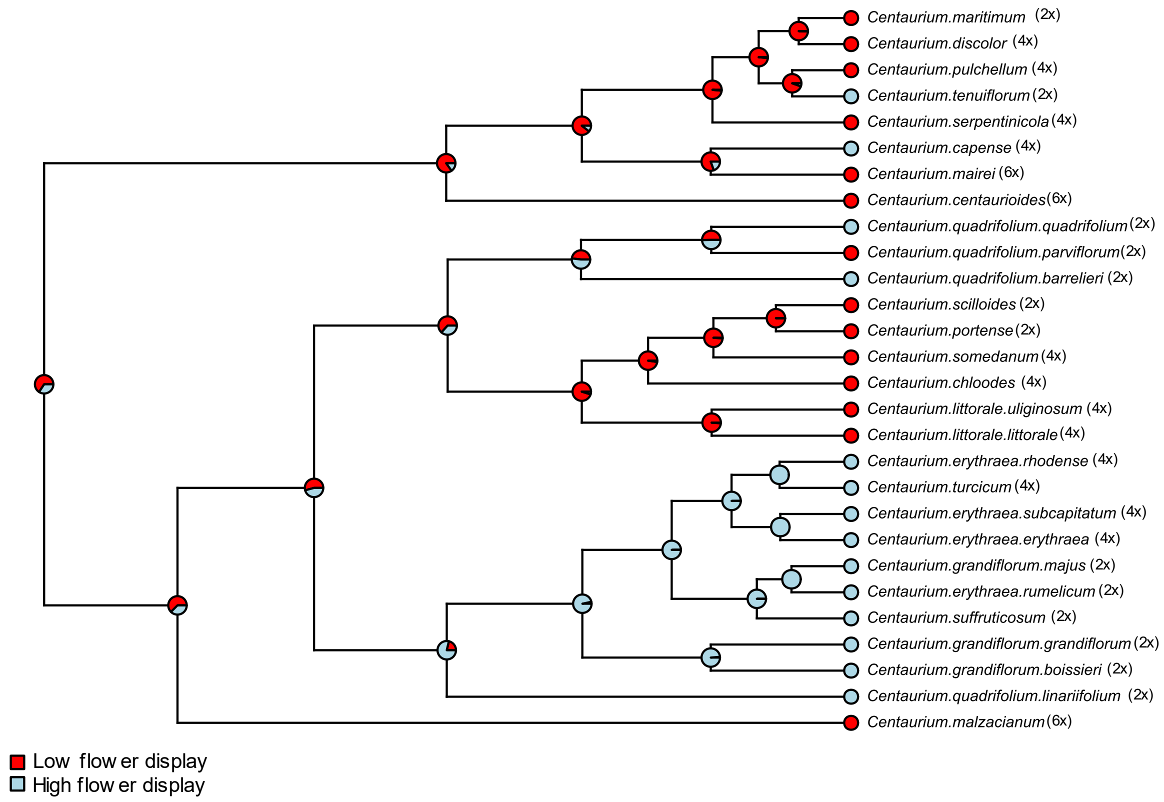
Centaurium.maritimum (2x)
Centaurium.discolor (4x)
Centaurium.pulchellum (4x)
Centaurium.tenuiflorum (2x)
Centaurium.serpentinicola (4x)
Centaurium.capense (4x)
Centaurium.mairei (6x)
Centaurium.centaurioides (6x)
Centaurium.quadrifolium.quadrifolium (2x)
Centaurium.quadrifolium.parviflorum (2x)
Centaurium.quadrifolium.barrelieri (2x)
Centaurium.scilloides (2x)
Centaurium.portense (2x)
Centaurium.somedanum (4x)
Centaurium.chloodes (4x)
Centaurium.littorale.uliginosum (4x)
Centaurium.littorale.littorale (4x)
Centaurium.erythraea.rhodense (4x)
Centaurium.turcicum (4x)
Centaurium.erythraea.subcapitatum (4x)
Centaurium.erythraea.erythraea (4x)
Centaurium.grandiflorum.majus (2x)
Centaurium.erythraea.rumelicum (2x)
Centaurium.suffruticosum (2x)
Centaurium.grandiflorum.grandiflorum (2x)
Centaurium.grandiflorum.boissieri (2x)
Centaurium.quadrifolium.linariifolium (2x)
Centaurium.malzacianum (6x)
Low flow er display
High flow er display

E)

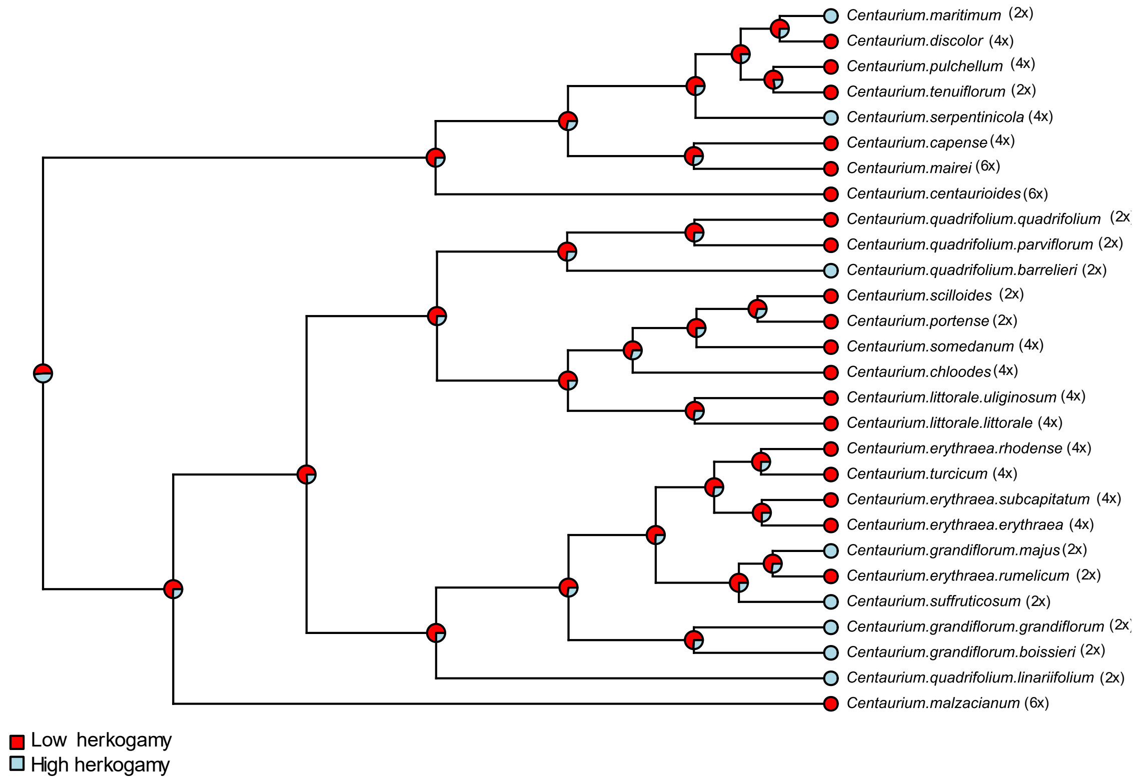
Centaurium.maritimum (2x)
Centaurium.discolor (4x)
Centaurium.pulchellum (4x)
Centaurium.tenuiflorum (2x)
Centaurium.serpentinicola (4x)
Centaurium.capense (4x)
Centaurium.mairei (6x)
Centaurium.centaurioides (6x)
Centaurium.quadrifolium.quadrifolium (2x
Centaurium.quadrifolium.parviflorum (2x)
Centaurium.quadrifolium.barrelieri (2x)
Centaurium.scilloides (2x)
Centaurium.portense (2x)
Centaurium.somedanum (4x)
Centaurium.chloodes (4x)
Centaurium.littorale.uliginosum (4x)
Centaurium.littorale.littorale (4x)
Centaurium.erythraea.rhodense (4x)
Centaurium.turcicum (4x)
Centaurium.erythraea.subcapitatum (4x)
Centaurium.erythraea.erythraea (4x)
Centaurium.grandiflorum.majus (2x)
Centaurium.erythraea.rumelicum (2x)
Centaurium.suffruticosum (2x)
Centaurium.grandiflorum.grandiflorum (2x)
Centaurium.grandiflorum.boissieri (2x)
Centaurium.quadrifolium.linariifolium (2x)
Centaurium.malzacianum (6x)
Low herkogamy
High herkogamy

F)

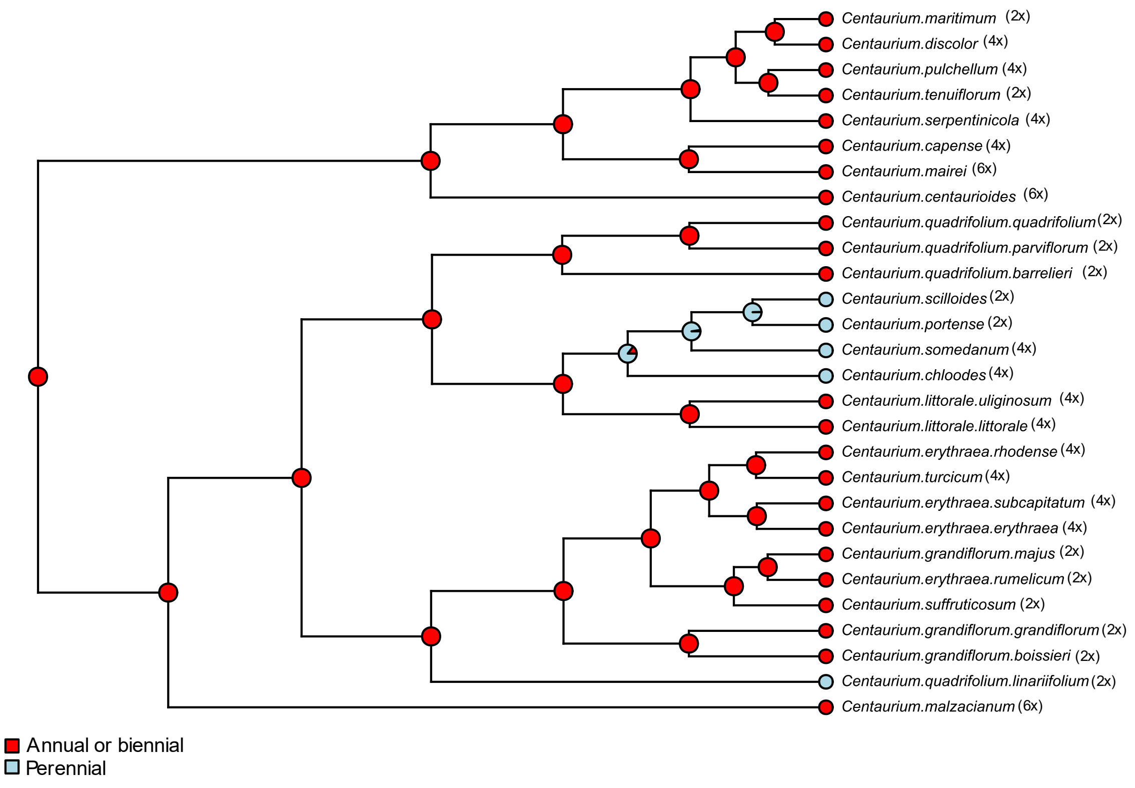

Centaurium.maritimum (2x)
Centaurium.discolor (4x)
Centaurium.pulchellum (4x)
Centaurium.tenuiflorum (2x)
Centaurium.serpentinicola (4x)
Centaurium.capense (4x)
Centaurium.mairei (6x)
Centaurium.centaurioides (6x)
Centaurium.quadrifolium.quadrifolium (2x)
Centaurium.quadrifolium.parviflorum (2x)
Centaurium.quadrifolium.barrelieri (2x)
Centaurium.scilloides (2x)
Centaurium.portense (2x)
Centaurium.somedanum (4x)
Centaurium.chloodes (4x)
Centaurium.littorale.uliginosum (4x)
Centaurium.littorale.littorale (4x)
Centaurium.erythraea.rhodense (4x)
Centaurium.turcicum (4x)
Centaurium.erythraea.subcapitatum (4x)
Centaurium.erythraea.erythraea (4x)
Centaurium.grandiflorum.majus (2x)
Centaurium.erythraea.rumelicum (2x)
Centaurium.suffruticosum (2x)
Centaurium.grandiflorum.grandiflorum (2x)
Centaurium.grandiflorum.boissieri (2x)
Centaurium.quadrifolium.linariifolium (2x)
Centaurium.malzacianum (6x)
Annual or biennial
Perennial
