# Supplementary material for: The interplay between climatic niche evolution, polyploidy and reproductive traits explains plant speciation in the Mediterranean Basin: a case study in Centaurium (Gentianaceae)
Source: Front Plant Sci. 2024 Aug 9;15:1439985. doi: 10.3389/fpls.2024.1439985 (PMC11344271; doi:10.3389/fpls.2024.1439985)
Supplement: Supplementary file 4 [file Table_1.pdf]

Table S1. Studied *Centaurium* taxa, ploidy level, chromosome number, number of records per taxa used in the climatic reconstruction of the genus, including political countries describing the natural distribution of the taxa and studied binary traits. The codification of the binary traits was as follows: diploid taxa were coded as “0” and tetra- and hexaploids as “1”; the length of the flower (flower size, FS) was coded as “0” (small- to-medium, < 15 mm) or “1” (large, > 15.1 mm); the flower display (FD) was the total number of flowers simultaneously present during anthesis and was coded as “0” (low number of flowers, < 30 flowers per plant) or “1” (high number of flowers, >30 flowers per plant); taxa with low herkogamy (Hk) were coded as “0” and the ones with high herkogamy as “1”. Annual or biennial species were coded as “0” and perennial ones as “1”.

Taxa	Ploidy level	Chromosome number (n)	Nº of records: political country (records/country)	Ploidy (binary)	FS	FD	Hk	LC
C. capense Broome	4x	18	43: Mexico (43)	1	0	1	0	1
C. centaurioides R.S. Rao & Hemadri	6x	28	21: India (21)	1	0	0	0	0
C. chloodes (Brot.) Samp.	4x	20	45: France (16), Portugal (8), Spain (21)	1	0	0	0	1
C. discolor (Gand.) Ronniger	4x	20	60: Spain	1	1	0	0	0
C. erythraea Rafn subsp. *erythraea*	4x	20	303: Austria (1), Belgium (2), Bulgaria (5), Denmark (2), France (62), Georgia (1), Germany (8), Greece (18), Hungary (2), Iran (1), Ireland (9), Israel (1), Italy (65), Poland (2), Portugal (5), Romania (11), Russia (5), Slovenia (1), Spain (43), Sweden (1), Switzerland (20), Turkey (20), United Kingdom (6), Yugoslavia (12)	1	0	1	0	0

C. erythraea subsp. *rhodense* (Boiss. & Reut.) Melderis	4x	20	55: Greece (10), Italy (22), Spain (10), Syria (1), Turkey (12)	1	0	1	0	0
C. erythraea subsp. *rumelicum* (Velen.) Melderis	2x	10	25: Algeria (2), France (5), Greece (5), Italy (2), Spain (2), Turkey (9)	0	0	1	0	0
C. erythraea var. *subcapitatum* (Corb.) Ubsdell	4x	20	8: France (4), Ireland (3), Spain (1)	1	0	1	0	0
C. grandiflorum subsp. *boissieri* (Willk.) Z. Díaz	2x	10	35: Spain (35)	0	1	1	1	0
C. grandiflorum (Pers.) Ronniger subsp. *grandiflorum*	2x	10	33: France (1), Spain (32)	0	1	1	1	0
C. grandiflorum subsp. *majus* (Hoffmanns. & Link) Z. Díaz	2x	10	75: Italy (14), Portugal (18), Spain (43)	0	1	1	1	0
C. littorale (Turner) Gilmour subsp. *littorale*	4x	20	81: Denmark (5), Estonia (56), Germany (2), Netherlands (11), Norway (1), Russia (1), Sweden (3), United Kingdom (2)	1	0	0	0	0
C. littorale subsp. *uliginosum* (Waldst. & Kit.) Melderis	4x	20	19: Austria (1), Denmark (4), France (1), Holand (1), Hungary (3), Sweden (2), Czechoslovakia (4), United Kingdom (3)	1	0	0	0	0
C. mairei Zeltner	6x	27	134: Algeria (18), Chypre (10), Egypt (15), India (4), Iran (63), Iraq (1), Israel (1), Jordan (5), Niger (2), North Yemen (3),	1	0	0	0	0

			Oman (4), Russia (1), Arabia Saudi (2), Senegal (1), Spain (1), Turkey (3)					
C. malzacianum Maire	6x	28	23: United Arab Emirates (1), Iran (3), North Yemen (8), Oman (11)	1	1	0	0	0
C. maritimum (L.) Fritsch	2x	10	48: Algeria (1), France (5), Greece (1), Italy (2), Morocco (1), Portugal (10), Spain (28)	0	1	0	1	0
C. portense Butcher	2x	10	7: Portugal (7)	0	1	0	0	1
C. pulchellum (Sw.) Druce	4x	18	380: Afganistan (2), Algeria (13), Armenia (1), Austria (1), Belgium (1), Bulgaria (1), China (26), Chypre (8), Crete (3), Denmark (1), Finland (1), France (21), Greece (29), Holand (1), Hungary (1), India (6), Iran (25), Israel (4), Italy (19), Morocco (19), Oman (4), Portugal (4), Rumania (4), Russia (12), Sardinia (1), Spain (97), Sweden (3), Syria (2), Czechoslovakia (1), Turkey (63), United Kingdom (3), Yugoslavia (3)	1	0	0	0	0
C. quadrifolium subsp. *barrelieri* (L.M. Dufour) G. López	2x	10	20: Spain (20)	0	1	1	1	0
C. quadrifolium subsp. *linariifolium* (Lam.) G. López	2x	10	12: Spain (12)	0	1	1	1	1

C. quadrifolium subsp. *parviflorum* (Willk.) Pedrol	2x	10	14: France (3), Spain (11)	0	0	0	0	0
C. quadrifolium subsp. *quadrifolium* (L.) G. López & C.E. Jarvis	2x	10	17: Spain (17)	0	0	1	0	0
C. scilloides (L. fil.) Samp.	2x	10	13: Portugal (9), Spain (4)	0	1	0	0	1
C. serpentinicola Carlström	4x	20	19: Greece (11), Turkey (8)	1	1	0	1	0
C. somedanum M. Laínz	4x	20	70: Spain (70)	1	1	0	0	1
C. suffruticosum (Griseb.) Ronniger	2x	10	29: Algeria (6), Morocco (16), Spain (7)	0	0	1	1	0
C. tenuiflorum (Hoffmanns. & Link) Fritsch	2x	20	77: Algeria (1), Chypre (1), Greece (7), Iran (2), Italy (3), Morocco (4), Portugal (9), Spain (40), Turkey (10)	0	0	1	0	0
C. turcicum (Velen.) Ronniger	4x	20	24: Algeria (1), Chypre (1), Greece (7), Iran (5), Italy (3), Morocco (4), Portugal (9), Russia (3), Spain (40), Turkey (28)	1	0	1	0	0
