# Supplementary material for: The interplay between climatic niche evolution, polyploidy and reproductive traits explains plant speciation in the Mediterranean Basin: a case study in Centaurium (Gentianaceae)
Source: Front Plant Sci. 2024 Aug 9;15:1439985. doi: 10.3389/fpls.2024.1439985 (PMC11344271; doi:10.3389/fpls.2024.1439985)
Supplement: Supplementary file 5 [file Table_2.pdf]

Table S2. Extended results of models of dependent and independent chromosome number changes correlated with floral size (*FS*), floral display (*FD*), herkogamy (*Hk*) and life cycle (*LC*). The best-fitted model, considering the Akaike's information criterion (*AIC*), is in bold font. *Df* indicates the calculated degrees of freedom and *LnLik* the log-likelihood. To study *FS*, taxa with small- to-medium flowers (< 15 mm) were coded as "1", and with large flowers (> 15.1 mm) as "2". Regarding *FD*, taxa with low number of flowers simultaneously present during anthesis (< 30 flowers per plant) were coded as "1", while the taxa having high number (>30 flowers per plant), as "2". Taxa with low herkogamy (*Hk*) were coded as "1" and the ones with high herkogamy as "2". Regarding life cycle (*LC*), annual or biennial species were coded as "1" and perennial as "2". Ascending (asc1, asc2) and descending (desc1, desc2) dysploidy and polyploidy (pol1, pol2) rates were estimated for traits 1 and 2. Transition rates from trait 1 to trait 2 and vice versa were also estimated (tran12, tran21).

Model	*Df*	*LnLik*	*AIC*	*asc1*	*desc1*	*asc2*	*desc2*	*pol1*	*pol2*	*tran12*	*tran21*
**dependent FS**	**10**	**-60.143**	**140.29**	**2.14E-07**	**8.49E+01**	**4.21E-08**	**1.17E-05**	**2.32E+00**	**2.79E+00**	**2.39E+00**	**1.41E+00**
independent FS	6	-66.316	144.63	9.45E-10	2.45E+00			1.65E+00		2.73E+00	2.22E+00
**dependent FD**	**10**	**-55.038**	**130.08**	**5.07E-06**	**5.96E-06**	**3.12E-08**	**3.61E+00**	**2.41E-01**	**4.39E+00**	**1.07E+00**	**6.53E-01**
independent FD	6	-62.897	137.79	1.05E-09	2.45E+00			1.65E+00		1.73E-01	8.93E-01
**dependent Hk**	**10**	**-58.429**	**136.86**	**1.13E-05**	**6.48E-08**	**4.57E-05**	**2.83E+00**	**4.84E-06**	**4.54E+00**	**2.11E+00**	**6.88E-01**
independent Hk	6	-65.03	142.06	3.28E-09	2.45E+00			1.65E+00		9.11E+01	3.64E+01
dependent LC	10	-55.002	130.00	7.07E-07	2.04E-07	1.89E-10	2.72E+00	2.42E+00	1.53E+00	7.46E-06	2.67E-01
**independent LC**	**6**	**-56.088**	**124.18**	**4.02E-12**	**2.45E+00**			**1.65E+00**		**2.56E-08**	**2.70E-01**
